# Can Vaccination Trigger Autoimmune Disorders? A Meta-Analysis

**DOI:** 10.3390/vaccines9080821

**Published:** 2021-07-25

**Authors:** Marek Petráš, Ivana Králová Lesná, Jana Dáňová, Alexander M. Čelko

**Affiliations:** 1 Department of Epidemiology and Biostatistics, Charles University in Prague-Third Faculty of Medicine, 100 00 Prague, Czech Republic; jana.danova@lf3.cuni.cz (J.D.); martin.celko@lf3.cuni.cz (A.M.Č.); 2 Laboratory for Atherosclerosis Research, Centre for Experimental Medicine, Institute for Clinical and Experimental Medicine, 140 21 Prague, Czech Republic; ivka@ikem.cz

**Keywords:** vaccine-associated autoimmune disease, immunization, meta-analysis, vaccine safety

## Abstract

Vaccination as an important tool in the fight against infections has been suggested as a possible trigger of autoimmunity over the last decades. To confirm or refute this assumption, a Meta-analysis of Autoimmune Disorders Association With Immunization (MADAWI) was conducted. Included in the meta-analysis were a total of 144 studies published in 1968–2019 that were available in six databases and identified by an extensive literature search conducted on 30 November 2019. The risk of bias classification of the studies was performed using the Newcastle–Ottawa Quality Assessment Scale. The strength of evidence was assessed using the Grading of Recommendations Assessment, Development, and Evaluation. While our primary analysis was conducted in terms of measures of association employed in studies with a low risk of bias, the robustness of the MADAWI outcome was tested using measures independent of each study risk of bias. Additionally, subgroup analyses were performed to determine the stability of the outcome. The pooled association of 0.99 (95% confidence interval, 0.97–1.02), based on a total of 364 published estimates, confirmed an equivalent occurrence of autoimmune disorders in vaccinated and unvaccinated persons. The same level of association reported by studies independently of the risk of bias was supported by a sufficient number of studies, and no serious limitation, inconsistency, indirectness, imprecision, and publication bias. A sensitivity analysis did not reveal any discrepancy in the primary result. Current common vaccination is not the cause of any of the examined autoimmune disorders in the medium and long terms.

## 1. Introduction

The commonly used vaccines are generally considered safe. However, as they stimulate the immune system, a legitimate question arises whether they can elicit, not only an immune, but also an autoimmune response.

As early as 1968, the first work appeared in which a case of no vaccine-associated multiple sclerosis was reported after vaccination against tuberculosis, tetanus, diphtheria, whooping cough, typhoid fever, polio, smallpox, or influenza [1]. It was not until 1994 that the first report on a causal relationship between several vaccines (e.g., diphtheria, tetanus or oral polio vaccine) and autoimmune diseases (e.g., Guillain–Barré syndrome, type 1 diabetes and multiple sclerosis) was published [2].

The number of autoimmune disorders (ADs) affecting at least 5% of individuals vaccinated in childhood has increased significantly within the last 30 years worldwide [3,4,5]. The question was even raised as to whether vaccination should or should not be recommended for those with a personal or family history of an AD [6].

The etiology and trigger mechanisms of ADs are still unclear [7]. Nevertheless, several studies have suggested that vaccination, as well as infection, could lead to the development of an AD in individuals with a genetic predisposition [8,9]. Hypotheses have been proposed that consider either molecular mimicry [7,10] or accidental activation of the host tissue self-antigens [11] as the main pathogenic mechanisms.

Numerous observational studies of varied risk of bias have demonstrated or refuted the concept of vaccine-associated ADs. Therefore, their results, expressed by measures of association (MAs), have been supported by several meta-analyses based on their summary association on the risk of each study bias. The weakness of these meta-analyses consists in the limited number of eligible studies, not allowing a generalization of their results.

Whether or not vaccination is in general able to influence the development of any AD was the goal of the present Meta-analysis of Autoimmune Disorders Association With Immunization (MADAWI). Hence, it was possible to include all studies in a quantitative analysis, regardless of the specificity of either the vaccine or AD. The sensitivity of the presumed association was further tested in various subgroups based on age, geographic region or year of publication, and others. We consider the MADAWI results crucial for improving the perception of vaccination by the general and professional public.

## 2. Materials and Methods

The conduct of the MADAWI followed the guidelines of the Preferred Reporting Items for Systematic Review and Meta-Analyses (PRISMA) [12] and Meta-analysis Of Observational Studies in Epidemiology (MOOSE) [13], which helped to identify eligible studies (Supplementary Materials).

Only controlled studies examining vaccine-associated ADs were eligible for inclusion in the MADAWI. A computerized search of the relevant literature was carried out in the following databases: Medical Literature Analysis and Retrieval System Online (MEDLINE), Excerpta Medica dataBASE (EMBASE), Derwent Drug File (DDFU), ProQuest Science & Technology (POSCITECH), BioSciences Information Service of Biological Abstracts (BIOSIS), and Chemical Abstracts Plus (HCAPLUS) from the earliest date available through to 30 November 2019, with no language restrictions.

The keywords were ‘vaccine’ and ‘immunization’ in combination with ‘controlled studies’ and ADs, including specific diseases and their synonyms (Supplementary Materials). A recursive search of references in full-text publications helped to identify additional articles beyond the computerized search.

Studies had to meet the following inclusion criteria: (1) controlled studies, such as a summary of clinical trials (SCT), case–control (C–C), and cohort (C) studies, including their modifications, i.e., self-controlled case-series (SCCS) or self-controlled risk interval (SCRI), as well as case cross-over (CCo) and case-centred (Cc) studies; (2) exposure to immunization with commonly used vaccines; (3) unvaccinated control group; and (4) MA, including the 95% confidence interval (CI).

Two reviewers independently extracted all relevant data using the following parameters: authors’ names; study period or year of publication; geographic region; participants, interventions, comparisons, outcomes and study design; time window; MA including 95% CI or raw data for odds ratio calculation; and factors of adjustment. If consensus was not achieved, the discrepancy was resolved through discussions among all authors.

To ensure unrepeated assignment of the MAs to the vaccine-associated ADs examined in one study, it was important to adopt a uniform procedure. Therefore, a selected MA had to be related to the general study population independently of age or region, and to the AD reported either any time or within the longest time gap post-vaccination. If only age- or region-specific MAs were identified, all of these were included in the MADAWI.

A study quality assessed by the risk of bias was based on the Newcastle–Ottawa Quality Assessment Scale (NOS) with a range of 0 to 9 stars [14]. The comparability, adequacy of cohort follow-up, as well as the same non-response rates of cases and controls assessed in the MADAWI are specified in Supplementary Materials. A summary association expressed by the pooled effect size (ES) was obtained from studies with a low risk of bias, and awarded at least 7 NOS stars. Irrespective of their risk of bias, all studies were used to assess the robustness of the primary outcome.

The strength of evidence for the primary outcome was based on the Grading of Recommendations Assessment, Development, and Evaluation (GRADE) guidelines [15]. The following criteria were adopted: (1) a sufficient number of MAs (≥10) [16] and >80% statistical power; (2) no serious limitation in low-risk studies; (3) no serious inconsistency of studies with an inconsistency index (I^2^) < 50% [17,18]; (4) no serious indirectness of evidence due to the high comparability of the patient and control groups in low-risk studies; (5) no serious imprecision of studies with a standard error < 0.1; and (6) no serious publication bias if the same association was found by fixed-effect and random-effects models [19]. In addition, the effects of small studies and absence of unpublished studies, or those not found, were also tested. The potential offset of future observations was estimated using a 95% prediction interval.

To accomplish the MADAWI objective, a null hypothesis was proposed to demonstrate the equivalence of AD occurrence in vaccinated versus unvaccinated individuals, i.e., H0: |ln (ES)| < 𝛿, where 𝛿 was the margin conventionally chosen to be 0.1, i.e., a 10% tolerance of disagreement, allowing an ES variance in the range from 0.91 to 1.10.

A conservative “combined” estimate of the pooled ES was applied. As homogeneity of the studies was not achieved, the outcome was assessed using the random-effects model (DerSimonian–Laird method; D–L) while the fixed-effect model (inverse variance method, I-V) helped to identify a possible publication bias.

The summary association was calculated based on the MAs and standard errors (SEs). Adjusted Mas, such as odds ratio (OR), relative risk (RR), hazard ratio (HR), or incidence rate ratio (IRR) were prioritized. The SEs were calculated based on the MAs and upper limits of 95% CI, as the limits were occasionally rounded to one decimal place. If an MA was not stated by the authors, crude OR and SEs were conventionally calculated based the study raw data.

The effect of small studies, determined using a meta-regression model with Egger’s test, demonstrated with significantly non-null bias coefficient [19]. The summary effect of asymmetry with the identification of any unpublished studies was estimated by the trim-and-fill method [19]. The prediction interval was calculated using the SEs of heterogeneity and the pooled ES [19]. The power of test was determined for a random-effects model [20,21,22].

Statistical analyses were performed using STATA version 15.1 (StataCorp. 2017. Stata Statistical Software: Release 15. College Station, TX, USA) at a significance level of α = 0.05 with a two-tailed 95% confidence interval.

This research was funded by UK PROGRES Q16—Environmental research project, Charles University, Prague, Czech Republic. The funder of the study had no role in study design, data collection, data analysis, data interpretation, or writing of the report.

## 3. Results

**Figure 1:**
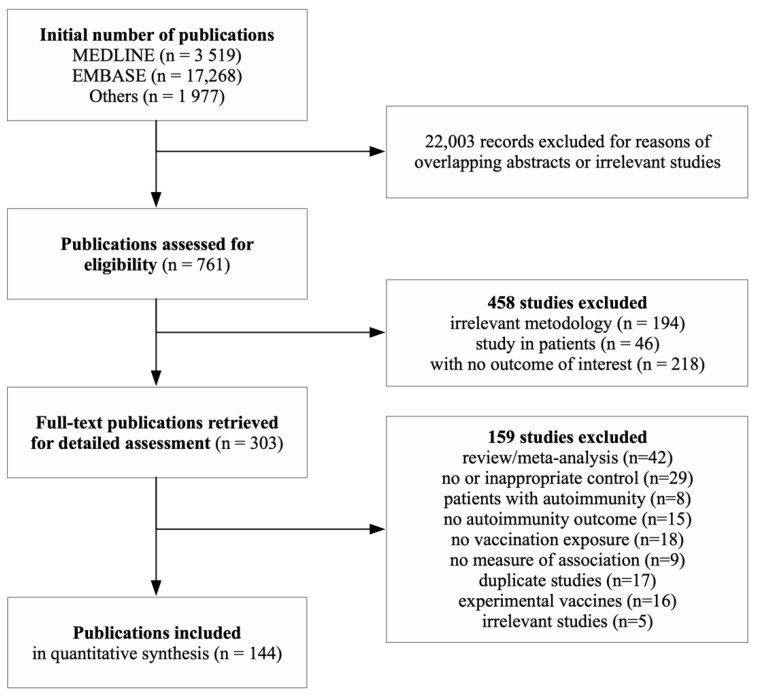
Flowchart.

A total of 22,764 publications were initially identified (Figure 1), of which 22,620 were excluded for reasons of irrelevant or duplicated studies and absent inclusion criteria. The remaining 144 eligible publications (Supplementary Materials) were published between 1968 and 2019 and reported a total of 562 unique MAs. The primary meta-analysis involved 364 MAs reported in 82 studies with a low risk of bias. The vaccine-associated ADs were assessed with adjusted HRs or RRs (43%), ORs (31%), and with crude ones (14%). The other MAs were calculated using raw data as crude ORs.

**Figure 2:**
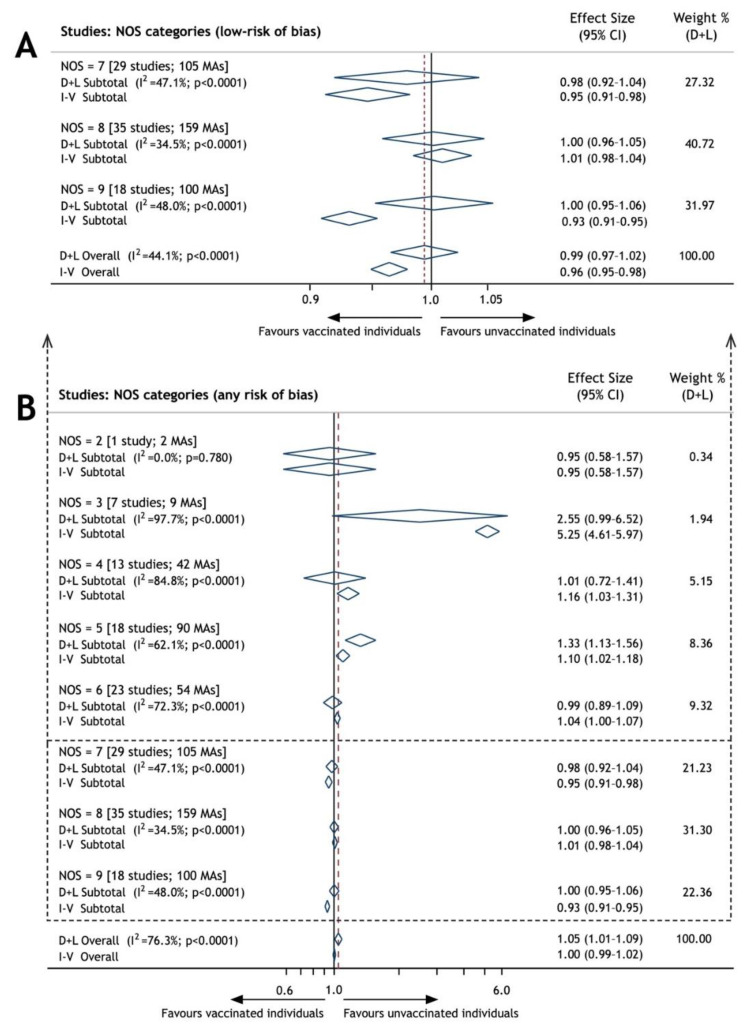
Forest plot of overall and NOS-grouped effect sizes of vaccine-associated autoimmune disorders; weight derived from random effect analysis (dashed line in point of pooled ES); (**A**) any risk of bias risk studies; (**B**) low-risk of bias studies; MAs: measures of association; NOS: Newcastle–Ottawa Scale; I^2^: index of inconsistency; D-L: DerSimonian–Laird method, random-effects model; and I-V: inverse variance method, fixed-effects model.

The pooled ES is depicted in Figure 2, including a sub-pooled ES for NOS-grouped studies. The primary summary association was estimated based on the results of the random-effects model, demonstrating no increased risk of vaccine-associated ADs, i.e., ES = 0.99 (95% CI: 0.97–1.02), *p* = 0.684. As both 95% CI limits laid within the range of equivalence, the null hypothesis was accepted.

The outcome met all the criteria required for assessing the strength of evidence (Table 1), i.e., a sufficient number of studies (82), including 364 MAs achieved a > 99% statistical power. The primary analysis conducted with low-risk studies was not burdened by any serious limitation and inconsistency (I^2^ = 44.1%), indirectness of evidence, imprecision (SE = 0.02) and no serious publication bias as demonstrated by the agreement of both associations obtained from the fixed-effect and random-effects models.

The effect of small studies assessed using the bias coefficient (0.27; 95% CI: 0.08–0.45), showed a shift towards vaccine-associated ADs. The trim-and-fill method revealed the absence of seven unpublished MAs that shifted the pooled ES (0.99; 95% CI: 0.96–1.02). Additionally, the 5% prediction interval with a narrow range of 0.73–1.36 suggested that any new ES estimation would not be far from the current result after the inclusion of data of future studies. These additional analyses thus confirmed that the summary outcome is consistent after counting unpublished or future studies, as well as after the elimination of small studies.

The pooled ES (1.05; 95% CI: 1.01–1.09) from all studies, irrespective of the risk of bias, confirmed the equivalence of ADs in the vaccinated versus unvaccinated individuals, thus supporting the robustness of the primary analysis outcome.

Consistent associations were obvious in the NOS-grouped studies with a low risk of bias, as well as in those with a still acceptable bias (NOS > 5), while vaccine-associated ADs were identified in moderate-risk studies, i.e., NOS = 5.

The impact of specific conditions on the outcome of interest was evaluated using sensitivity tests with MAs grouped by location of the AD, vaccine type, population age, study area, year of study publication, and study methodology. Out of a total of 50 ADs, 47 included in the primary meta-analysis (except for inflammatory and psoriatic polyarthritis and glomerulonephritis, which were reported only in studies with moderate- or high-risk of bias). The ADs were arranged into eight location-classified groups (Table 2). As inactivated and live vaccines can differ in their mechanism of action, the MAs were arranged according to the type of vaccine: inactivated, live, and lipid-adjuvanted vaccines. Furthermore, vaccines employed in unspecific immunization or of ambiguous type were assessed in a group of unspecified vaccines.

Whether the pooled ES can be sensitive to the study methodology was examined in the groups of C–C and C studies, as well as in modified studies based on self-controlled principles. The impact of possible confounders on the pooled outcome was tested in the group of crude and adjusted MAs. The occurrence of age-specific ADs has been investigated in several studies; hence, the MAs were grouped by children and adolescents, adults, and the general population. Overall, the studies were conducted in different countries across four continents: Europe, North America, Asia, and Australia. Whether the primary outcome can be influenced by the study area was tested by quantitative analyses in subgroups of studies assigned to continents. A possible time-dependence of MAs was assessed in subgroups of low-risk studies arranged by four periods by the year of publication, i.e., ≤1990, 1991–2000, 2001–2010, and ≥2011.

Consistent with the primary outcome, sensitivity tests conducted in the above groups and sub-groups of low-risk studies identified no vaccine-associated ADs. Despite this, the absent robustness of several associations suggested a possible effect of studies with moderate- or high-risk of bias (Table 3).

## 4. Discussion

The MADAWI clearly demonstrated no increase in AD occurrence associated with the commonly used vaccines, thus confirming our hypothesis of equivalent AD incidence in both the vaccinated and unvaccinated individuals, a finding previously reported in studies with both low-risk and any-risk of bias. Furthermore, the outcome can be generalized as all criteria supporting the strength of evidence were met. Likewise, additional analyses demonstrated the stability of the summary association with regard to the effect of small, unpublished, and/or future studies. The independence of ADs from vaccination was not affected by the age- or area-specific study populations, year of publication or study methodology.

The unique approach of the MADAWI based on the assessment of any vaccine-associated AD helped to produce a robust outcome outbalancing the insufficient number of studies with a low risk of bias in previous meta-analyses. However, it is true that our literature search identified three meta-analyses investigating the potential development of any AD after vaccination against human papillomavirus (HPV) [23,24] or hepatitis B virus (HBV) [25] whose summary associations were in line with our conclusion.

A specific meta-analysis, focused on a group of endocrine ADs, showed no relationship with immunization, regardless of the type of vaccine used. The same conclusion was drawn in a meta-analysis assessing the incidence of type I diabetes in children after their vaccination against tuberculosis, tetanus, diphtheria, whooping cough, *Haemophilus influenza* type b, poliomyelitis or measles, mumps, and rubella [26].

Likewise, no association was documented between neurological ADs, including demyelinating diseases involving the central nervous system (i.e., acute disseminated encephalomyelitis or acquired demyelinating syndrome, multiple sclerosis, and/or optic neuritis), and immunization with any type of vaccine. This result was in accordance with those of previous meta-analyses or reviews addressing demyelinating diseases of the central nervous system [27,28] and multiple sclerosis [29] after HBV or HPV vaccination [30,31]. Furthermore, no increased risk of multiple sclerosis or optic neuritis developing after vaccination with inactivated and live vaccines has been reported by any other review [32] or quantitative analysis [33]. It has even been found that vaccination against tetanus or diphtheria can reduce the incidence of multiple sclerosis or demyelinating diseases compared to unvaccinated persons [33,34]. Unfortunately, this result could not be confirmed or refuted due to the insufficient number of low-risk studies, and the outcome could be burdened by moderate or high bias.

A slightly increased incidence of Guillain–Barré syndrome associated with a trivalent inactivated influenza vaccine was documented in a meta-analysis that included studies conducted in the 1981–2014 period [35]. When conducting a similar meta-analysis of only low-risk studies performed in the 1981–2019 period, the occurrence of Guillain–Barré syndrome was not higher in vaccinated against versus unvaccinated individuals [36].

Likewise, the MADAWI did not demonstrate an increased frequency of gastrointestinal ADs after any vaccination, and a similar conclusion was reported by a quantitative analysis assessing the incidence of inflammatory bowel disease (IBD) of autoimmune nature in children vaccinated with various childhood vaccines [37]. By contrast, while children vaccinated against poliomyelitis were not at an increased risk of developing IBD, the incidence rates of Crohn’s disease and ulcerative colitis were slightly higher than those found in unvaccinated children. The authors suggested this may have been due to the high heterogeneity of the small number of studies included in their meta-analysis.

Furthermore, vaccination did not increase the incidence of autoimmune skin diseases or vasculitis, as shown by subgroup analyses of the MADAWI, a finding consistent with those of previous meta-analyses of studies focused on skin ADs or vasculitis after HPV vaccination [23,24]. Similarly, no relationship between Kawasaki syndrome and rotavirus vaccination was reported in a review complemented with a meta-analysis [38].

Given that the MADAWI focused on the development of an AD at any time or within the longest time gap post-vaccination, the outcome of our primary analysis should be interpreted in the context of ADs of medium or long duration. The MADAWI results cannot be easily extrapolated to short-term ADs that may resolve spontaneously within 2–6 months post-vaccination. A typical example of these ADs is immune thrombocytopenia documented after vaccination with a monovalent or combined measles vaccine that disappeared spontaneously in 93% of children, with no risk of developing its long-lasting or chronic form [39]. Therefore, the validity of the MADAWI outcome may be limited to the potential development of early and short-term ADs post-vaccination.

## 5. Conclusions

The conclusions of not only the MADAWI, but also previous meta-analyses clearly show that common vaccines for routine or on-demand immunization cannot be considered a cause or a trigger of ADs of medium- or long-term persistence independently of the time gap after vaccination. Although the MADAWI results are robust and unambiguous, they may not be generally applicable to new genetic or experimental vaccines, as well as to ADs whose relationship to vaccination has not been studied and/or reported to date.

## Figures and Tables

**Table 1:** Overall and NOS-grouped effect sizes, including parameters of the strength of evidence, robustness, and prediction interval.

Studies’ NOS ^1^-Scaled Groups	Pooled ES ^2^ (95% CI ^3^) *	No Serious Publication Bias (Pooled ES; 95% CI) **	95% Prediction Interval	Number of MAs ^4^	No Serious Studies’ Inconsistency (I^2^) ^5^	No Serious Impression (SE) ^6^	Power of Test (%)	Robustness
**2**	0.95 (0.58–1.57)	Yes (0.95; 0.58–1.57)	0.02–39.1	2	Yes (0.0%)	No (0.25)	-	NA ^8^
**3**	2.55 (0.99–6.52)	No (5.25; 4.61–5.97)	0.03–194	9	No (97.7%)	No (0.48)	43.3	NA
**4**	1.01 (0.72–1.41)	No (1.16; 1.03–1.31)	0.10–10.0	43	No (84.8%)	No (0.17)	8.5	NA
**5**	1.33 (1.13–1.56)	Yes (1.10; 1.02–1.18)	0.43–4.14	90	No (62.1%)	Yes (0.08)	83.0	NA
**6**	0.99 (0.89–1.09)	No (1.04; 1.01–1.07)	0.59–1.66	54	No (72.3%)	Yes (0.05)	92.0	NA
**7**	0.98 (0.92–1.04)	Yes (0.95; 0.91–0.98)	0.63–1.51	105	Yes (47.1%)	Yes (0.03)	99.3	NA
**8**	1.00 (0.96–1.05)	Yes (1.01; 0.98–1.04)	0.74–1.36	159	Yes (34.5%)	Yes (0.02)	>99.9	NA
**9**	1.00 (0.95–1.06)	Yes (0.93; 0.91–0.95)	0.72–1.40	100	Yes (48.0%)	Yes (0.03)	99.6	NA
**Overall, any RoB ^7^**	1.05 (1.01–1.09)	No (1.00; 0.99–1.02)	0.55–1.99	562	No (76.3%)	Yes (0.02)	90.7	NA
**Overall, low RoB**	0.99 (0.97–1.02)	Yes (0.96; 0.95–0.98)	0.73–1.36	364	Yes (44.1%)	Yes (0.02)	>99.9	Yes

^1^ NOS: Newcastle–Ottawa Scale; ^2^ ES: effect size, ^3^ CI: confidence interval; ^4^ MAs: measures of association; ^5^ I^2^: index of inconsistency; ^6^ SE: standard deviation; ^7^ RoB: risk of bias; ^8^ NA: not applicable; *: pooled effect size derived from the random-effects model including 95% confidence interval; and **: pooled effect size derived from the fixed-effects model including 95% confidence interval.

**Table 2:** Examined autoimmune disorders classified to location.

Location	Autoimmune Disorders
Endocrine	Addison’s disease, Graves–Basedow’s disease, Hashimoto’s thyroiditis, Hyperthyroidism, Hypothyroidism, Pernicious anemia, Type 1 diabetes, Thyroiditis
Neurological	Acute disseminated encephalomyelitis, Acquired/Acute demyelinating syndrome, Bell’s palsy, Guillain–Barré syndrome, Myasthenia gravis, Multiple sclerosis, Optic neuritis, Narcolepsy
Gastrointestinal	Autoimmune hepatitis, Celiac disease, Crohn’s disease, Inflammatory bowel disease, Pancreatitis, Ulcerative colitis
Haematological	Autoimmune haemolytic anemia, Immune thrombocytopenia/Idiopathic thrombocytopenic purpura
Dermatological	Alopecia areata, Psoriasis, Vitiligo
Connective tissue	Ankylosing spondylitis, Sjögren’s syndrome, Inflammatory polyarthritis, Juvenile arthritis, Myositis, Psoriatic arthritis, Rheumatoid arthritis, Scleroderma (local and/or systemic), Systemic lupus
Vasculitis	Behcet’s disease, Erythema nodosum, Henoch–Schönlein purpura, Kawasaki syndrome, Polyarteritis nodosa, Vasculitis, Wegener’s granulomatosis
Others	Glomerulonephritis, Raynaud’s syndrome, Uveitis

**Table 3:** Subgroup analysis of effect size, including parameters of strength of evidence, robustness, and prediction interval.

Subgroups	Pooled ES ^1^ (95% CI ^2^) *	No Serious Publication Bias (Pooled ES; 95% CI) **	95% Prediction Interval	Number of MAs ^3^	No Serious Studies’ Inconsistency (I^2^) ^4^	No Serious Impression (SE) ^5^	Power of Test (%)	Robustness (Pooled ES; 95% CI) †
**Autoimmune disorders**								
Endocrine	0.96 (0.92–1.00)	Yes (0.97; 0.95–1.00)	0.72–1.29	120	Yes (45.6%)	Yes (0.02)	90.0	Yes (0.93; 0.89–0.98)
Neurological	1.02 (0.95–1.10)	Yes (0.91; 0.89–0.94)	0.65–1.60	95	Yes (47.0%)	Yes (0.04)	73.0	No (1.10; 1.02–1.20)
Gastrointestinal	1.03 (0.93–1.13)	No (1.06; 1.00–1.12)	0.63–1.66	41	No (50.9%)	Yes (0.05)	40.3	Yes (1.05; 0.96–1.14)
Hematologic	1.12 (0.91–1.36)	Yes (1.09; 0.97–1.24)	0.43–2.89	27	No (52.4%)	No (0.10)	5.2	No (1.22; 1.02–1.47)
Dermatological	0.98 (0.85–1.13)	Yes (0.98; 0.87–1.11)	0.68–1.41	10	Yes (7.9%)	Yes (0.07)	17.5	Yes (1.28; 0.86–1.90)
Connective tissue	0.98 (0.93–1.04)	Yes (0.98; 0.93–1.04)	0.87–1.10	47	Yes (<0.1%)	Yes (0.03)	5.0	Yes (1.07; 0.97–1.19)
Vasculitis	1.09 (0.91–1.29)	Yes (1.08; 0.94–1.25)	0.60–1.96	20	Yes (22.6%)	Yes (0.09)	5.3	Yes (1.12; 0.96–1.31)
**Type of vaccine**								
Killed	1.00 (0.97–1.03)	Yes (1.00; 0.98–1.02)	0.73–1.36	251	Yes (38.0%)	Yes (0.02)	>99.9	No (1.07; 1.01–1.12)
Live	0.99 (0.92–1.07)	Yes (0.94; 0.90–0.98)	0.60–1.62	72	No (54.4%)	Yes (0.04)	90.5	Yes (1.00; 0.93–1.07)
Unspecific	1.03 (0.93–1.15)	Yes (0.90; 0.87–0.93)	0.73–1.47	19	Yes (41.4%)	Yes (0.06)	41.2	Yes (1.09; 0.95–1.25)
Lipid-adjuvants	0.94 (0.83–1.07)	Yes (0.95; 0.85–1.06)	0.63–1.41	22	Yes (14.5%)	Yes (0.07)	68.3	Yes (0.96; 0.86–1.06)
**Study methodology**								
Case–control	0.96 (0.91–0.99)	Yes (0.91; 0.89–0.93)	0.74–1.22	152	Yes (21.6%)	Yes (0.02)	>99.9	Yes (1.03; 0.98–1.09)
Cohort	1.00 (0.95–1.04)	Yes (0.98; 0.95–1.00)	0.67–1.47	176	Yes (49.4%)	Yes (0.02)	>99.9	Yes (1.05; 0.97–1.13)
Modified	1.08 (0.99–1.16)	Yes (1.03; 0.99–1.07)	0.82–1.41	36	Yes (26.9%)	Yes (0.04)	11.6	No (1.11; 1.01–1.22)
**Measures of association**								
Crude	0.97 (0.90–1.05)	Yes (0.97; 0.92–1.02)	0.60–1.56	94	Yes (35.7%)	Yes (0.04)	96.4	Yes (1.07; 0.98–1.18)
Adjusted	1.00 (0.97–1.03)	Yes (0.96; 0.95–0.98)	0.74–1.36	270	Yes (46.7%)	Yes (0.02)	>99.9	Yes (1.04; 0.99–1.08)
**Age of study population**								
<18 years	1.02 (0.98–1.06)	Yes (1.02; 0.99–1.04)	0.78–1.32	184	Yes (27.3%)	Yes (0.02)	99.9	Yes (1.00; 0.95–1.04)
>18 years	0.99 (0.94–1.05)	Yes (0.96; 0.93–1.00)	0.65–1.50	91	No (56.3%)	Yes (0.03)	98.7	Yes (1.06; 0.99–1.13)
General population	0.95 (0.90–1.02)	Yes (0.90 (0.88–0.93)	0.65–1.39	89	Yes (42.3%)	Yes (0.03)	>99.9	Yes (1.09; 0.99–1.21)
**Study locality**								
Asia	1.09 (0.94–1.26)	Yes (1.09; 0.94–1.26)	0.80–1.48	16	Yes (<0.1%)	Yes (0.08)	5.0	Yes (1.28; 0.84–1.94)
Australia	1.10 (0.86–1.41)	Yes (1.10; 0.86–1.41)	0.63–1.95	6	Yes (<0.1%)	No (0.13)	5.0	Yes (0.92; 0.77–1.09)
Europe	1.01 (0.98–1.04)	Yes (0.97; 0.95–0.99)	0.76–1.33	229	Yes (41.2%)	Yes (0.02)	>99.9	Yes (1.02; 0.98–1.06)
North America	0.96 (0.90–1.02)	Yes (0.92; 0.89–0.96)	0.58–1.57	113	No (53.0%)	Yes (0.03)	99.9	Yes (1.07; 0.99–1.15)
**Year of publication**								
1990 or older	1.04 (0.83–1.31)	Yes (1.04; 0.83–1.31)	0.62–1.75	7	Yes (<0.1%)	No (0.12)	5.0	Yes (0.88; 0.75–1.03)
1991–2000	0.98 (0.91–1.06)	Yes (0.98; 0.92–1.05)	0.77–1.25	43	Yes (9.6%)	Yes (0.04)	87.5	Yes (0.93; 0.86–1.01)
2001–2010	1.00 (0.93–1.08)	Yes (0.99; 0.94–1.04)	0.63–1.58	73	Yes (44.5%)	Yes (0.04)	82.5	Yes (1.04; 0.97–1.11)
2011 or newer	1.00 (0.96–1.03)	Yes (0.96; 0.94–0.98)	0.72–1.37	241	Yes (48.5%)	Yes (0.02)	>99.9	No (1.09; 1.03–1.15)

^1^ ES: effect size, ^2^ CI: confidence interval; ^3^ MAs: measures of association; ^4^ I^2^: index of inconsistency; ^5^ SE: standard deviation; *: pooled effect size derived from the random-effects model including 95% confidence interval; **: pooled effect size derived from the fixed-effects model including 95% confidence interval; and †: pooled effect size derived from the random-effects model including 95% confidence interval (studies of any risk of bias).

## Data Availability

Not applicable.

## Supplementary Materials

The following are available online at https://www.mdpi.com/article/10.3390/vaccines9080821/s1. Table S1: MOOSE Checklist, Table S2: Search strategy, Table S3: Studies included into MADAWI, Table S4: Descriptive information about and quality assessment of included studies.
