# Supplementary material for: Can Vaccination Trigger Autoimmune Disorders? A Meta-Analysis
Source: Vaccines (Basel). 2021 Jul 25;9(8):821. doi: 10.3390/vaccines9080821 (PMC8402438; doi:10.3390/vaccines9080821)
Supplement: Supplementary file 1 [file vaccines-09-00821-s001.zip › vaccines-1279255-supplementary.pdf]

Supplementary

Table S1. MOOSE Checklist.

Criteria	Brief description
**Introduction**	
Problem definition	Autoimmune disorders may be caused or triggered by vaccination.
Hypothesis statement	Null hypothesis: equivalence of autoimmune disorders occurrence in vaccinated versus unvaccinated individual.
A statement of objectives that includes the study population, condition of interest, exposure or intervention, and the outcome(s) considered	Study outcome: autoimmune disorders<br>Study exposure: vaccination<br>Study population: general (any age, any geographic region)
**Sources**	
Qualifications of literature searchers (e.g., librarians and investigators)	The credential of the only literature researcher (MF) is available in the Acknowledgments section.
Search strategy including the time period required for the synthesis and keywords	The search strategy is detailed in Appendix, Table 2S.
Effort to include all available studies, including contact with authors	References of all retrieved articles and recent reviews were reviewed.
Databases and registries searched	Medical Literature Analysis and Retrieval System Online (MEDLINE), Excerpta Medica dataBASE (EMBASE), Derwent Drug File (DDFU), ProQuest Science & Technology (POSCITECH), BioSciences Information Service of Biological Abstracts (BIOSIS), and Chemical Abstracts Plus (HCAPLUS)
Search software used, name and version, including special features used (e.g., explosion)	No special search software was used.
Use of hand searching (e.g., reference lists of eligible articles)	References of all retrieved articles and recent reviews were reviewed.
List of citations located and those excluded, including justification	Details of the literature search process are outlined in the flow chart.
Method of addressing articles published in languages other than English	No language restrictions.
Method of handling abstracts and unpublished studies	The search process was not restricted to peer-reviewed studies.
Description of any contact with authors	Not done.
**Study selection**	
Types of study designs considered	Pooled clinical trials, case-control and cohort studies, self-controlled case-series, self-controlled risk interval, case cross-over, case-centered studies
Relevance or appropriateness of studies gathered for assessing the hypothesis to be tested	(1) controlled studies; (2) exposure to immunization with commonly used vaccines; (3) unvaccinated control group; and (4) outcome of interest determined by the measures of association, including the 95% confidence interval.
Rationale for the selection and coding of data (eg, sound clinical principles or convenience)	Study characteristics were extracted independently by two investigators (MP and IKL). Measures of associations (incl. 95% CI) for autoimmune disorders and vaccinations were assessed separately whenever possible. In cases where a study reported more than one measure of association of autoimmune disorders or vaccinations, each autoimmune disorder and each vaccination were extracted separately. The most adjusted measure of association was included when a study reported more than one measure.
Documentation of how data were classified and coded (e.g., multiple raters, blinding, and interrater reliability)	Data were independently extracted and analyzed by two investigators (MP and JD) with the final decision reached by consensus.
Assessment of confounding (e.g., comparability of cases and controls in studies where appropriate)	Table 4S, study groups comparability assessed using the Newcastle-Ottawa Quality Assessment Scale and adjustment of association estimations. Although the adjustments varied across studies, at least sex or age was always applied for adjustment.
Assessment of study quality, including blinding of quality assessors: stratification or regression on possible predictors of study results	The quality of each study was assessed by two investigators (MP and JD) using the Newcastle-Ottawa Quality Assessment Scale (NOS). NOS-based assessment was different for cohort and case-control studies. The derived self-controlled case-series, self-controlled risk interval or clinical trials were assigned to the group of cohort studies assessment, and the case-crossover or case-centered studies to the case-control ones.

	Full comparability of the cohorts or AD patients and controls was achieved if they matched in sex and age as well as in at least another arbitrary parameter. Adequacy of cohorts' follow-up as well as the same non-response rates of cases and controls were adopted if not exceeding a 20% of difference.
Assessment of heterogeneity	The Q-statistic and I-squared statistic were used to assess the heterogeneity among studies.
Statistical methods (e.g., complete description of fixed or random effects models, justification of whether the chosen models account for predictors of study results, dose-response models, or cumulative meta-analysis) in sufficient detail to be replicated	Description of the methods of meta-analyses, subgroup analyses, and assessment of publication bias are detailed in the "Statistical methods" and "Results" section.
**Results**	
A graph summarizing individual study estimates and the overall estimate	A forest plot of subtotal results in groups of studies according to NOS scale is incorporated in the main body of text (Figure 2). Conservative forest plots cannot be displayed because of the high number of measures of association. One main table (Table 1) and one supplemental table (Table 3) are provided.
A table giving descriptive information for each study included	Table 4S
Results of sensitivity testing (e.g., subgroup analysis)	See "Results" section; Table 3
Indication of statistical uncertainty of findings	95% confidence intervals are provided with all summary effect estimates.
**Discussion**	
Strengths and weaknesses	The strength of evidence; primary analysis included only studies with low-risk bias; additional analyzes of effect of small and unpublished studies.
Potential biases in the review process (e.g., publication bias)	See "Statistical Methods", "Results" and "Discussion" section.
Justification for exclusion (e.g., exclusion of non-English-language citations)	See "Material and methods" section
Assessment of quality of included studies	Studies have been subgroup analyzed by the quality.
Consideration of alternative explanations for observed results	The pooled outcome of the primary analysis must be interpreted for ADs of medium- or long-term persistence because they were included at any time or within the longest time gap after vaccination.
Generalization of the conclusions (i.e., appropriate for the data presented and within the domain of the literature review)	The summary outcome can be generally accepted because all criteria supporting the strength of evidence were met.
Guidelines for future research	We underlined that our findings cannot be extrapolated to short-term autoimmune disorders resolving spontaneously within 2–6 months. Moreover, the findings are not applicable to new genetic or experimental vaccines that require additional observational studies.
Disclosure of funding source	This research was funded by UK PROGRES Q16- Environmental research project, Charles University in Prague, Czech Republic.

Table S2. Search strategy.

Criteria
L1 QUE ?IMMUN? OR ?IMUN? OR ?INFLAMMAT?
Words for autoimmune illness in general:
L2 QUE AUTOIMMUN? OR AUTO(W)IMMUN? OR AUTOIMUN? OR AUTO(W)IMUN? OR AUTOINFLAMMAT? OR AUTO(W)INFLAMMAT?
Words for vaccination:
L3 QUE ?VACCIN? OR IMMUNI!ATION OR ?IMUNI!ATION OR ?IMMUNI!ATION OR ?IMMUNI!ED OR ?IMUNI!ED
Words for cohort studies/clinical trials:
L5 QUE COHORT? OR CASE(W)CONTROL? OR CLINICAL(W)(TRIAL OR STUD?) OR RANDOMI?(4A)(CONTROL? OR CLINIC? OR TRIAL OR STUD?)
Words for the negation of animals:
L6 QUE ANIMAL OR CAT OR DOG OR RABBIT OR GUINEA PIG OR MOUSE OR MICE OR RAT OR CHICKEN OR DUCK OR POULTRY OR GOAT OR SHEEP OR PIG OR PORCINE OR SWINE OR HORSE OR CATTLE OR MONKEY OR RAM OR FISH
Endocrine system illnesses

L8 QUE (L1 OR HASHIMOTO## OR ATROPHIC)(2A)?THYROID? OR (L1 OR TYPE(W)1 OR INSULIN(W)DEPENDENT OR JUVENILE)(3A)DIABETES OR (L1 OR PRIMARY) (A)ADRENAL(W)INSUFFI? OR HYPOADRENOCORTIC? OR HYPO(W)ADRENOCORTIC?
L9 QUE (POLYGLANDULAR OR SCHMIDT##)(W)SYNDROM## OR TYPE(2A)APS OR L1(W)POLYENDOCRINOPATHY(W)CANDIDIASIS(W)ECTODERMAL(W) DYSPLASIA OR PREMATUR?(A)(OVARIAN(W)FAILURE OR MENOPAUSE)
L10 QUE L1(S)(ENDOCRINOPAT? OR GONADAL? OR INFERTIL? OR PITUITARY? OR HYPOPHYSIS OR PARATHYROID?)
L11 QUE (HASHIMOTO## OR GRAVE## OR BASEDOW## OR GRAVE##(A)BASEDOW## OR ADDISON##)(2A)(SYNDROM## OR DISEASE)
Autoimmune thyroid disease, autoimmune thyroiditis, Hashimoto´s thyroiditis, atrophic thyroiditis, chronic autoimmune thyroiditis, Diabetes mellitus type 1, Type 1 diabetes, insulin-dependent diabetes mellitus, juvenile diabetes, latent autoimmune diabetes of adults, autoimmune diabetes, Autoimmune adrenal insufficiency, primary adrenal insufficiency , Addison´s disease, Hypoadrenocorticism , autoimmune polyglandular syndroms (APS), Type I APS, autoimmune polyendocrinopathy candidiasis ectodermal dysplasia (APECED), Type II APS, Schmidt´s syndrome, Type III APS, Premature ovarian failure, Premature menopause, primary ovarian insufficiency, gonadal autoimmunity, immunological infertility, Pituitary autoimmunity, parathyroid autoimmunity, Grave´s disease, toxic diffuse goiter, Basedow disease
Nervous system illnesses
L13 QUE ?ENCEPHALIT? OR ?ENCEPHALOPAT? OR ?MYELITIS OR L1(3A)MYELOPATH?
L14 QUE MULTIPLE(W)SCLEROSIS
L15 QUE DEMYELINAT? OR OPTIC?(W)NEURITIS OR MYASTHENIA(W)GRAVIS OR (L1 OR ACQUIRED)(2A)NEUROMYOTONIA OR (DIFUSE OR DIFFUSE)(W)CEREBRAL
L16 QUE (L1 OR PERIPHERAL OR VASCULIT? OR IGA OR MULTIFOCAL)(2A)NEUROPATHY
L17 QUE (GUILLAIN(W)BARRE OR ISAAC## OR DEVIC## OR DEGO###)(2A)(SYNDROM## OR DISEASE)
L18 QUE (NEUROLOGICAL(W)EVENT OR NARCOLEPSY OR PARESIS OR VISUAL LOSS OR FACIAL PARALYSIS OR HEADACHE OR BLURRED VISION OR DOUBLE VISION OR DIPLOPIA OR SLEEP DISORDER)
encephalomyelitis, Autoimmune encephalopathies, acute disseminated encephalomyelitis, acute haemorrhagic leuco-encephalitis, haemorrhagic leuco-encephalitis, chronic progressive inflammatory myelopathy, myelitis, transverse myelitis, neuromyelitis optica (Devic´s syndrome), multiple sclerosis, Demyelinating diseases, demyelination, central demyelination, Acute demyelinating multiple sclerosis , optic neuritis, Myasthenia gravis, acquired neuromyotonia (Isaac´s disease), diffuse cerebral , peripheral neuropathy, chronic inflammatory demyelinating polyneuropathy, Vasculitic neuropathy, Multifocal Motor Neuropathy (MMN) or MMNCB, Guillain-Barré syndrome, Degos´s disease, Neurological event, Narcolepsy, Paresis, visual loss, facial paralysis, headache, blurred vision, diplopia, Sleep disorders
Musculosceletal/systemic illnesses
L21 QUE CONNECTIVE(W)TISSUE(W)DISEASE OR ANKYLOSING(W)SPONDYLITIS OR HENOCH(W)(SCHONLEIN## OR SCHOENLEIN##)(2W)PURPURA
L22 QUE (L1 OR RHEUMATOID OR JUVENILE OR PSORIATIC)(W)ARTHRITIS OR POLYMYALGIA(W)RHEUMATICA OR LUPUS
L23 QUE (CHRONIC(W)FATIGUE OR OVERLAP)(W)SYNDROM##
L24 QUE FIBROMYALGIA OR (SJORGEN## OR SJOERGEN## OR BEHCET##)(2A)(SYNDROM## OR DISEASE)
L25 QUE (?MYOSITIS NOT POLIOMYOSITIS)
Connective tissue diseases, Undifferentiated/undetermined connective tissue disease, mixed connective tissue disease, Ankylosing spondylitis, Henoch-Schonlein´s purpura, rheumatoid artrhritis, juvenile chronic arthritis, juvenile idiopatic arthritis, chronic inflammatory arthritis, Psoriatic arthritis, polymyalgia rheumatica, systemic lupus erythematosus , chronic fatigue syndrome, overlap syndrome, Myositis not POLIOMYOSITIS, polymyositis, dermatomyositis, idiopathic inflammatory myopathies, fibromyalgia , Sjorgen´s syndrome, Behcet´s syndrome
Vasculitis
L27 QUE L1(S)VASCULIT? OR (POLYANGIITIS OR WEGENER## OR EOSINOPHILIC)(2A)GRANULOMATOSIS OR POLYMYALGIA(W)RHEUMATICA
L28 QUE (KAWASAKI OR CHURG(A)STRAUSS OR TAKAYASU## OR AORTIC ARCH OR MUCOCUTANEOUS LYMPH NODE)(2A)(SYNDROM## OR DISEASE)
L29 QUE ?ARTERITIS(A)(TAKAYASU## OR NODOS? OR MICROSCOPIC OR GIANT(W)CELL OR TEMPORAL) OR MICROSCOPIC(W)POLYANGIITIS
Vasculitis , urticarial vasculitis, vasculitus, Eosinophilic granulomatosis with polyangiitis (EGPA), Wegener´s granulomatosis, Granulomatosis with polyangiitis, Churg-Strauss syndrome, Kawasaki syndrome, mucocutaneous lymph node syndrome, Takayasu´s disease, Takayasu's arteritis, aortic arch syndrome, microscopic polyarteritis, Microscopic polyangiitis, polyarteritis nodosa, giant-cell arteritis, Temporal arteritis, polymyalgia rheumatica
ASIA syndrome
L7 QUE L1(S)ASIA OR L1(W)(SYNDROM## OR DISEASE)(5A)ADJUVANS OR ASIA(2A)SYNDROM##
autoimmune/autoinflammatory syndrome induced by adjuvans ASIA
Words for illnesses of heart/lung/kidney
L31 QUE L1(S)(?CARDITI? OR ?MYOPATH?)
L32 QUE L1(S)?FIBROSIS OR FIBROSING(W)ALVEOLITIS OR LYMPH?(W)INTERSTITI?(A)PNEUMON?
L33 QUE (L1 OR NECROTI?)(W)(GLOMERULONEPHR? OR GLOMERUL?(W)NEPHR? OR IGA(W)NEPHROP?) OR L1(S)CRYOGLOBULIN?
L34 QUE ((ANTIGLOMERULAR OR ANTI(W)GLOMERULAR)(W)BASEMENT(W)MEMBRANE OR ANTI(W)GBM OR ANTI(W)TBM OR GOODP!STURE## OR BERGER)(2A)(SYNDROM## OR DISEASE OR NEPHRIT?)

heart: Carditis, Autoimmune myocarditis, Recurrent pericarditis, autoimmune pericarditis, autoimmune cardiomyopathy
lung: Fibrosis , fibrosing alveolitis, Idiopathic pulmonary fibrosis, cryptogenic fibrosing alveolitis, Lymphoid interstitial pneumonitis, Lymphocytic interstitial pneumonia
kidney:Autoimmune glomerulonephritis, glomerular nephritis, necrotizing glomerulonephritis, immune complex glomerulonephritis, IgA nephropathy, Berger's disease, mixed essential cryoglobulinaemia, cryoglobulinaemia, cryoglobulinemic disease, anti-GBM disease, antiglomerular basement membrane disease, Goodpasture´s syndrome, Anti-GBM nephritis, Anti-TBM nephritis
GIT illnesses
L36 QUE L1(2A)ENTEROPATHY OR (CELIAC OR COELIAC OR GLUTEN)(2A)(ENTEROPATHY OR DISEASE OR SPRUE)
L37 QUE L1(S)(COLITIS OR HEPATITIS OR PANCREATITIS OR ILEITIS) OR CROHN##(2A)ILEITIS
L38 QUE INFLAMMATORY(W)BOWEL(W)DISEASE OR (PRIMARY(W)BILIARY OR SCLEROS?)(W)(CIRRHOSIS OR CHOLANGITIS)
L39 QUE (CROHN## OR IPEX)(2A)(SYNDROM## OR DISEASE)
Autoimmune enteropathy , IPEX syndrome, celiac disease, coeliac-like enterophathy, gluten enterophathy, ulcerative colitis, Colitis , inflammatory bowel disease, Primary biliary cirrhosis, Primary biliary cholangitis, sclerosing cholangitis, autoimmune hepatitis, lupoid hepatitis, autoimmune pancreatitis, Ileitis , Crohn's ileitis, Crohn disease
Hematologic illnesses
L41 QUE L1(S)(?CYTOPENI? OR NEUTROPENIA) OR THROMBOCYTOPEN?(2W)PURPURA OR (L1 OR HAEMOLYTIC OR HEMOLYTIC OR APLASTIC)(2W)(ANAEMIA)
L42 QUE (ANTIPHOSPHOLIPID? OR ANTI(W)PHOSPHOLIPID?)(2A)(SYNDROM## OR DISEASE)
Cytopenia , Immune thrombocytopenia, autoimmune thrombocytopenia, idiopatic thrombocytopenic purpura, thrombotic thrombocytopenic purpura, autoimmune haemolytic anaemia, aplastic anaemia, Pernicious anemia (PA), autoimmune neutropenia, Anti-phospholipid syndrome
Illness of skin/eye
L44 QUE ERYTHEMA(W)NODOSUM OR SCLEROSIS OR SCLERODERMA OR PSORIASIS OR VITILIGO OR (ALOPECIA OR BALDNESS OR HAIR LOSS)
L45 QUE BULLOUS(W)DERMATOS? OR L1(S)BULLOUS OR PEMPHIGUS OR PEMPHIGOID OR DERMATITIS(W)HERPETIFORMIS
L46 QUE UVEITIS
Skin: Erythema nodosum, systemic sclerosis, localized scleroderma, Systemic scleroderma, diffuse scleroderma, psoriasis, vitiligo, alopecia areata, bulous dermatoses, Autoimmune bullous disease, pemphigus vulgaris, pemphigus foliaceus, bullous pemphigoid, pemphigoid, dermatitis herpetiformis
Eye: Uveitis
Illness list X (rare illnesses)
L48 QUE (STILL## OR BALO OR COGAN## OR CREST OR EVANS OR LAMBERT(W)EATON OR PARRY(W)ROMBERG OR PARSONAGE(W)TURNER OR RAYNAUD## OR SUSAC## OR TOLOSA(W)HUNT OR ORMOND##)(3A)(SYNDROM## OR DISEASE)
L49 QUE (VOGT(W)KOYANAGI(W)HARADA OR COLD(W)AGGLUTININ OR IGG4(W)RELATED OR LINEAR(W)IGA OR STIFF(W)(PERSON OR MAN))(3A)(SYNDROM## OR DISEASE) OR BALO(A)SCLEROS?
L50 QUE EOSINOPHILIC(W)FASCIITIS OR HIDRADENITIS(W)SUPPURATIVA OR ACNE(W)INVERSA OR MOOREN##(W)ULCER OR PALINDROMIC(W)RHEUMATISM OR PAROXYSMAL(W)NOCTURNAL(W)(HEMOGLOBINURIA OR HAEMOGLOBINURIA)
L51 QUE PARS(W)PLANITIS OR PROGESTERONE(W)DERMATITIS OR PURE(W)RED(W)CELL(W)APLASIA OR PYODERMA(W)GANGRENOSUM OR RAYNAUD##(W)PHENOMENON OR RELAPSING(W)POLYCHONDRITIS
L52 QUE REFLEX(W)SYMPATHETIC(W)DYSTROPHY OR COMPLEX(W)REGIONAL(W)PAIN OR RETROPERITONEAL(W)FIBROSIS OR RHEUMATIC(W)FEVER OR SARCOIDOSIS OR SCLERITIS OR SUBACUTE(W)BACTERIAL(W)ENDOCARDITIS
Adult Still's disease , Baló disease, Balo concentric sclerosis, Cogan’s syndrome, Cold agglutinin disease, CREST syndrome, Evans syndrome, IgG4-related sclerosing disease, IgG4-related disease, Lambert-Eaton syndrome , Linear IgA disease, Parry Romberg syndrome, Parsonage-Turner syndrome, POEMS syndrome, Stiff person syndrome (SPS), Susac’s syndrome, Tolosa-Hunt syndrome (THS), Vogt-Koyanagi-Harada Disease, Eosinophilic fasciitis, Hidradenitis Suppurativa, Acne Inversa, Mooren’s ulcer, Palindromic rheumatism, Paroxysmal nocturnal hemoglobinuria (PNH), Pars planitis (peripheral uveitis), Progesterone dermatitis, Pure red cell aplasia (PRCA), Pyoderma gangrenosum, Raynaud’s phenomenon , Reflex sympathetic dystrophy, Complex Regional Pain Syndrome, Relapsing polychondritis, Retroperitoneal fibrosis, Ormond's disease, Rheumatic fever, Sarcoidosis, Scleritis, Subacute bacterial endocarditis (SBE)
Skin: Erythema nodosum, systemic sclerosis, localized scleroderma, Systemic scleroderma, diffuse scleroderma, psoriasis, vitiligo, alopecia areata, bulous dermatoses, Autoimmune bullous disease, pemphigus vulgaris, pemphigus foliaceus, bullous pemphigoid, pemphigoid, dermatitis herpetiformis

Table S3. Studies included into MADAWI.

Study	Reference
Abbasi 2017	Abbasi M, Nabavi SM, Fereshtehnejad SM, Jou NZ, Ansari I, Shayegannejad V, Mohammadianinejad SE, Farhoudi M, Noorian A, Razazian N, Abedini M, Faraji F. Multiple sclerosis and environmental risk factors: a case-control study in Iran. Neurol Sci. 2017 Nov;38(11):1941-1951.
Ahlgren 2009	Ahlgren C, Toren K, Oden A, Andersen O. A population- based case-control study on viral infections and vaccinations and subsequent multiple sclerosis risk. European Journal of Epidemiology 2009;24(9):541–52.
Alter 1968	Alter M, Speer J. Clinical evaluation of possible etiologic factors in multiple sclerosis. Neurology 1968;18:109–116.
Altobelli 2003	Altobelli E, Petrocelli R, Verrotti A, Valenti M. Infections and risk of type I diabetes in childhood: a population-based case-control study. Eur J Epidemiol. 2003;18(5):425-30.
Andersen 1981	Andersen E, Isager H, Hyllested K. Risk factors in multiple sclerosis: tuberculin reactivity, age at measles infection, tonsillectomy and appendectomy. Acta Neurol Scand. 1981 Feb;63(2):131-5.
Andrews 2017	Andrews N, Stowe J, Miller E. No increased risk of Guillain-Barré syndrome after human papilloma virus vaccine: A self-controlled case-series study in England. Vaccine. 2017 Mar 23;35(13):1729-1732.
Angelo 2014	Angelo MG, David MP, Zima J, Baril L, Dubin G, Arellano F, Struyf F. Pooled analysis of large and long-term safety data from the human papillomavirus-16/18-AS04-adjuvanted vaccine clinical trial programme. Pharmacoepidemiol Drug Saf. 2014 May;23(5):466-79.
Arnheim-Dahlström 2013	Arnheim-Dahlström L, Pasternak B, Svanström H, Sparén P, Hviid A. Autoimmune, neurological, and venous thromboembolic adverse events after immunisation of adolescent girls with quadrivalent human papillomavirus vaccine in Denmark and Sweden: cohort study. BMJ. 2013 Oct 9;347:f5906.
Ascherio 2001	Ascherio A, Zhang SM, Hernan M A, Olek MJ, Coplan PM, Brodovicz K, Walker A. M. Hepatitis B vaccination and the risk of multiple sclerosis. N Engl J Med 2001;344 (5), 327–332.
Baker 2019	Baker MA, Baer B, Kulldorff M, Zichittella L, Reindel R, DeLuccia S, Lipowicz H, Freitas K, Jin R, Yih WK. Kawasaki disease and 13-valent pneumococcal conjugate vaccination among young children: A self-controlled risk interval and cohort study with null results. PLoS Med. 2019 Jul 2;16(7):e1002844.
Bansil 1997	Bansil S, Singhal BS, Ahuja GK, Riise T, Ladiwala U, Behari M, Cook SD. Multiple sclerosis in India: a case-control study of environmental exposures. Acta Neurol Scand. 1997 Feb;95(2):90-5.
Bardenheier 2016	Bardenheier BH, Duffy J, Duderstadt SK, Higgs JB, Keith MP, Papadopoulos PJ, Gilliland WR, McNeil MM. Anthrax Vaccine and the Risk of Rheumatoid Arthritis and Systemic Lupus Erythematosus in the U.S. Military: A Case-Control Study. Mil Med. 2016 Oct;181(10):1348-1356.
Baron 2005	Baron S, Turck D, Leplat C, Merle V, Gower-Rousseau C, Marti R, Yzet T, Lerebours E, Dupas JL, Debeugny S, Salomez JL, Cortot A, Colombel JF. Environmental risk factors in paediatric inflammatory bowel diseases: a population based case control study. Gut. 2005 Mar;54(3):357-63.
Baxter 2013	Baxter R, Bakshi N, Fireman B, Lewis E, Ray P, Vellozzi C, Klein NP. Lack of association of Guillain-Barré syndrome with vaccinations. Clin Infect Dis. 2013 Jul;57(2):197-204.
Baxter 2016	Baxter R, Lewis E, Fireman B, DeStefano F, Gee J, Klein NP. Case-centered Analysis of Optic Neuritis After Vaccines. Clin Infect Dis. 2016 Jul 1;63(1):79-81.
Belongia 2010	Belongia EA, Irving SA, Shui IM, Kulldorff M, Lewis E, Yin R, Lieu TA, Weintraub E, Yih WK, Li R, Baggs J; Vaccine Safety Datalink Investigation Group. Real-time surveillance to assess risk of intussusception and other adverse events after pentavalent, bovine-derived rotavirus vaccine. Pediatr Infect Dis J. 2010 Jan;29(1):1-5.
Bengtsson 2010	Bengtsson C, Kapetanovic MC, Källberg H, Sverdrup B, Nordmark B, Klareskog L, Alfredsson L; EIRA Study Group. Common vaccinations among adults do not increase the risk of developing rheumatoid arthritis: results from the Swedish EIRA study. Ann Rheum Dis. 2010 Oct;69(10):1831-3.
Bernstein 2007	Bernstein CN, Rawsthorne P, Blanchard JF. Population-based case-control study of measles, mumps, and rubella and inflammatory bowel disease. Inflamm Bowel Dis. 2007 Jun;13(6):759-62.
Berr 1989	Berr C, Puel J, Clanet M, Ruidavets JB, Mas JL, Alperovitch A. Risk factors in multiple sclerosis: a population-based case-control study in Hautes-Pyrénées, France. Acta Neurol Scand. 1989 Jul;80(1):46-50.
Bertuola 2010	Bertuola F, Morando C, Menniti-Ippolito F, Da Cas R, Capuano A, Perilongo G, Da Dalt L. Association between drug and vaccine use and acute immune thrombocytopenia in childhood: a case-control study in Italy. Drug Saf. 2010 Jan 1;33(1):65-72.
Beyerlein 2017	Beyerlein A, Strobl AN, Winkler C, Carpus M, Knopff A, Donnachie E, Ankerst DP, Ziegler AG. Vaccinations in early life are not associated with development of islet autoimmunity in type 1 diabetes high-risk children: Results from prospective cohort data. Vaccine. 2017 Mar 27;35(14):1735-1741.
Black 2002	Black SB, Lewis E, Shinefield HR, Fireman B, Ray P, DeStefano F, Chen R. Lack of association between receipt of conjugate haemophilus influenzae type B vaccine (HbOC) in infancy and risk of type 1 (juvenile onset) diabetes: long term follow-up of the HbOC efficacy trial cohort. Pediatr Infect Dis J. 2002 Jun;21(6):568-9.
Black 2003	Black C, Kaye JA, Jick H. MMR vaccine and idiopathic thrombocytopaenic purpura. Br J Clin Pharmacol. 2003 Jan;55(1):107-11.

Block 2010	Block SL, Brown DR, Chatterjee A, Gold MA, Sings HL, Meibohm A, Dana A, Haupt RM, Barr E, Tamms GM, Zhou H, Reisinger KS. Clinical trial and post-licensure safety profile of a prophylactic human papillomavirus (types 6, 11, 16, and 18) l1 virus-like particle vaccine. Pediatr Infect Dis J. 2010 Feb;29(2):95-101.
Blom 1991	Blom L, Nyström L, Dahlquist G. The Swedish childhood diabetes study. Vaccinations and infections as risk determinants for diabetes in childhood. Diabetologia. 1991 Mar;34(3):176-81.
Cardwell 2008	Cardwell CR, Carson DJ, Patterson CC. No association between routinely recorded infections in early life and subsequent risk of childhood-onset Type 1 diabetes: a matched case-control study using the UK General Practice Research Database. Diabet Med. 2008 Mar;25(3):261-7.
Casetta 1994	Casetta I, Granieri E, Malagù S, Tola MR, Paolino E, Caniatti LM, Govoni V, Monetti VC, Fainardi E. Environmental risk factors and multiple sclerosis: a community-based, case-control study in the province of Ferrara, Italy. Neuroepidemiology. 1994;13(3):120-8.
Compston 1986	Compston DA, Vakarelis BN, Paul E, McDonald WI, Batchelor JR, Mims CA. Viral infection in patients with multiple sclerosis and HLA-DR matched controls. Brain. 1986 Apr;109 (Pt 2):325-44.
Cooper 2002	Cooper GS, Dooley MA, Treadwell EL, St Clair EW, Gilkeson GS. Risk factors for development of systemic lupus erythematosus: allergies, infections, and family history. J Clin Epidemiol 2002;55(10):982–9.
Da Dalt 2016	Da Dalt L, Zerbinati C, Strafella MS, Renna S, Riceputi L, Di Pietro P, Barabino P, Scanferla S, Raucci U, Mores N5, Compagnone A, Da Cas R, Menniti-Ippolito F7; Italian Multicenter Study Group for Drug and Vaccine Safety in Children. Henoch-Schönlein purpura and drug and vaccine use in childhood: a case-control study. Ital J Pediatr. 2016 Jun 18;42(1):60.
Dauvilliers 2013	Dauvilliers Y, Arnulf I, Lecendreux M, Monaca Charley C, Franco P, Drouot X, d'Ortho MP, Launois S, Lignot S, Bourgin P, Nogues B, Rey M, Bayard S, Scholz S, Lavault S, Tubert-Bitter P, Saussier C, Pariente A; Narcoflu-VF study group. Increased risk of narcolepsy in children and adults after pandemic H1N1 vaccination in France. Brain. 2013 Aug;136(Pt 8):2486-96.
Davis 2001	Davis RL, Kramarz P, Bohlke K, Benson P, Thompson RS, Mullooly J, Black S, Shinefield H, Lewis E, Ward J, Marcy SM, Eriksen E, Destefano F, Chen R; Vaccine Safety Datalink Team. Measles-mumps-rubella and other measles-containing vaccines do not increase the risk for inflammatory bowel disease: a case-control study from the Vaccine Safety Datalink project. Arch Pediatr Adolesc Med. 2001 Mar;155(3):354-9.
Deceuninck 2018	Deceuninck G, Sauvageau C, Gilca V, Boulianne N, De Serres G. Absence of association between Guillain-Barré syndrome hospitalizations and HPV-vaccine. Expert Rev Vaccines. 2018 Jan;17(1):99-102.
DeStefano 2001	DeStefano F, Mullooly JP, Okoro CA, Chen RT, Marcy SM, Ward JI, Vadheim CM, Black SB, Shinefield HR, Davis RL, Bohlke K; Vaccine Safety Datalink Team. Childhood vaccinations, vaccination timing, and risk of type 1 diabetes mellitus. Pediatrics. 2001 Dec;108(6):E112.
DeStefano 2003	DeStefano F, Verstraeten T, Jackson LA, Okoro CA, Benson P, Black SB, Shinefield HR, Mullooly JP, Likosky W, Chen RT. Vaccinations and risk of central nervous system demyelinating diseases in adults. Arch Neurol. 2003 Apr;60(4):504-9.
Duderstadt 2012	Duderstadt SK, Rose CE Jr, Real TM, Sabatier JF, Stewart B, Ma G, Yerubandi UD, Eick AA, Tokars JI, McNeil MM. Vaccination and risk of type 1 diabetes mellitus in active component U.S. Military, 2002-2008. Vaccine. 2012 Jan 17;30(4):813-9.
Eftekharian 2014	Eftekharian MM, Mousavi M, Hormoz MB, Roshanaei G, Mazdeh M. Multiple sclerosis and immunological-related risk factors: results from a case-control study. Hum Antibodies. 2014;23(1-2):31-6.
EURODIAB 2000	EURODIAB Substudy 2 Study Group. Infections and vaccinations as risk factors for childhood type I (insulin-dependent) diabetes mellitus: a multicentre case-control investigation. EURODIAB Substudy 2 Study Group. Diabetologia. 2000 Jan;43(1):47-53.
Feeney 1997	Feeney M, Ciegg A, Winwood P, Snook J. A case-control study of measles vaccination and inflammatory bowel disease. The East Dorset Gastroenterology Group. Lancet. 1997 Sep 13;350(9080):764-6.
France 2008	France EK, Glanz J, Xu S, Hambidge S, Yamasaki K, Black SB, Marcy M, Mullooly JP, Jackson LA, Nordin J, Belongia EA, Hohman K, Chen RT, Davis R; Vaccine Safety Datalink Team. Risk of immune thrombocytopenic purpura after measles-mumps-rubella immunization in children. Pediatrics. 2008 Mar;121(3):e687-92.
Frisch 2018	Frisch M, Besson A1, Clemmensen KKB, Valentiner-Branth P, Mølbak K, Hviid A. Quadrivalent human papillomavirus vaccination in boys and risk of autoimmune diseases, neurological diseases and venous thromboembolism. Int J Epidemiol. 2018 Apr 1;47(2):634-641.
Galeotti 2013	Galeotti F, Massari M, D'Alessandro R, Beghi E, Chiò A, Logroscino G, Filippini G, Benedetti MD, Pugliatti M, Santuccio C, Raschetti R; ITANG study group. Risk of Guillain-Barré syndrome after 2010-2011 influenza vaccination. Eur J Epidemiol. 2013 May;28(5):433-44.
Garbe 2012	Garbe E, Andersohn F, Bronder E, Salama A, Klimpel A, Thomae M, Schrezenmeier H, Hildebrandt M, Späth-Schwalbe E, Grüneisen A, Meyer O, Kurtal H. Drug-induced immune thrombocytopaenia: results from the Berlin Case-Control Surveillance Study. Eur J Clin Pharmacol. 2012 May;68(5):821-32.
Geier 2005	Geier DA, Geier MR. A case-control study of serious autoimmune adverse events following hepatitis B immunization. Autoimmunity. 2005 Jun;38(4):295-301.
Geier 2017	Geier DA, Geier MR. Quadrivalent human papillomavirus vaccine and autoimmune adverse events: a case-control assessment of the vaccine adverse event reporting system (VAERS) database. Immunol Res. 2017 Feb;65(1):46-54.
Gilat 1987	Gilat T, Hacohen D, Lilos P, Langman MJ. Childhood factors in ulcerative colitis and Crohn's disease. An international cooperative study. Scand J Gastroenterol. 1987 Oct;22(8):1009-24.

Glatthaar 1988	Glatthaar C, Whittall DE, Welborn TA, Gibson MJ, Brooks BH, Ryan MM, Byrne GC. Diabetes in Western Australian children: descriptive epidemiology. Med J Aust. 1988 Feb 1;148(3):117-23.
Graves 1999	Graves PM, Barriga KJ, Norris JM, Hoffman MR, Yu L, Eisenbarth GS, Rewers M. Lack of association between early childhood immunizations and beta-cell autoimmunity. Diabetes Care. 1999 Oct;22(10):1694-7.
Greene 2012	Greene SK, Rett M, Weintraub ES, et al. Risk of confirmed Guillain-Barré syndrome following receipt of monovalent inactivated influenza A (H1N1) and seasonal influenza vaccines in the Vaccine Safety Datalink Project, 2009–2010. Am J Epidemiol 2012 Jun 1;175(11): 1100–09.
Grimaldi-Bensouda 2011	Grimaldi-Bensouda L, Alpérovitch A, Besson G, Vial C, Cuisset JM, Papeix C, Lyon-Caen O, Benichou J, Rossignol M; Lucien Abenhaim for the GBS-PGRx Study Group. Guillain-Barre syndrome, influenzalike illnesses, and influenza vaccination during seasons with and without circulating A/H1N1 viruses. Am J Epidemiol. 2011 Aug 1;174(3):326-35.
Grimaldi-Bensouda 2012	Grimaldi-Bensouda L, Michel M, Aubrun E, Leighton P, Viallard JF, Adoue D, Magy-Bertrand N, Tisserand G, Khellaf M, Durand JM, Quittet P, Fain O, Bonnotte B, Morin AS, Limal N, Costedoat-Chalumeau N, Morel N, Pan-Petesch B, Decaux O, Mahevas M, Ruel M, Sacre K, Lefrere F, Abenhaim L, Godeau B. A case-control study to assess the risk of immune thrombocytopenia associated with vaccines. Blood. 2012 Dec 13;120(25):4938-44.
Grimaldi-Bensouda 2014	Grimaldi-Bensouda L, Guillemot D, Godeau B, Bénichou J, Lebrun-Frenay C, Papeix C, Labauge P, Berquin P, Penfornis A, Benhamou PY, Nicolino M, Simon A, Viallard JF, Costedoat-Chalumeau N, Courcoux MF, Pondarré C, Hilliquin P, Chatelus E, Foltz V, Guillaume S, Rossignol M, Abenhaim L. Autoimmune disorders and quadrivalent human papillomavirus vaccination of young female subjects. J Intern Med. 2014 Apr;275(4):398-408.
Grimaldi-Bensouda 2017	Grimaldi-Bensouda L, Rossignol M, Koné-Paut I, Krivitzky A, Lebrun-Frenay C, Clet J, Brassat D, Papeix C, Nicolino M, Benhamou PY, Fain O, Costedoat-Chalumeau N, Courcoux MF, Viallard JF, Godeau B, Papo T, Vermersch P, Bourgault-Villada I, Breart G, Abenhaim L. Risk of autoimmune diseases and human papilloma virus (HPV) vaccines: Six years of case-referent surveillance. J Autoimmun. 2017 May;79:84-90.
Grimaldi-Bensouda A 2014	Grimaldi-Bensouda L, Le Guern V, Kone-Paut I, Aubrun E, Fain O, Ruel M, Machet L, Viallard JF, Magy-Bertrand N, Daugas E, Rossignol M, Abenhaim L, Costedoat-Chalumeau N. The risk of systemic lupus erythematosus associated with vaccines: an international case-control study. Arthritis Rheumatol. 2014 Jun;66(6):1559-67.
Gronlund 2016	Grönlund O, Herweijer E, Sundström K, Arnheim-Dahlström L. Incidence of new-onset autoimmune disease in girls and women with pre-existing autoimmune disease after quadrivalent human papillomavirus vaccination: a cohort study. J Intern Med. 2016 Dec;280(6):618-626.
Hansen 2011	Hansen TS, Jess T, Vind I, Elkjaer M, Nielsen MF, Gamborg M, Munkholm P. Environmental factors in inflammatory bowel disease: a case-control study based on a Danish inception cohort. J Crohns Colitis. 2011 Dec;5(6):577-84.
Hapfelmeier 2019	Hapfelmeier A, Gasperi C, Donnachie E, Hemmer B. A large case-control study on vaccination as risk factor for multiple sclerosis. Neurology. 2019 Aug 27;93(9):e908-e916.
Harrison 1997	Harrison BJ, Thomson W, Pepper L, Ollier WE, Chakravarty K, Barrett EM, Silman AJ, Symmons DP. Patients who develop inflammatory polyarthritis (IP) after immunization are clinically indistinguishable from other patients with IP. Br J Rheumatol. 1997 Mar;36(3):366-9.
Hernįn 2004	Hernán MA, Jick SS, Olek MJ, Jick H. Recombinant hepatitis B vaccine and the risk of multiple sclerosis: a prospective study. Neurology. 2004 Sep 14;63(5):838-42.
Ho 2012	Ho TY, Huang KY, Huang TT, Huang YS, Ho HC, Chou P, Lin CH, Wei CK, Lian WC, Chen TC, Huang HB, Lee CC. The impact of influenza vaccinations on the adverse effects and hospitalization rate in the elderly: a national based study in an Asian country. PLoS One. 2012;7(11):e50337.
Hughes 2006	Hughes RA, Charlton J, Latinovic R, Gulliford MC. No association between immunization and Guillain-Barré syndrome in the United Kingdom, 1992 to 2000. Arch Intern Med. 2006 Jun 26;166(12):1301-4.
Hviid 2004	Hviid A, Stellfeld M, Wohlfahrt J, Melbye M. Childhood vaccination and type 1 diabetes. N Engl J Med. 2004 Apr 1;350(14):1398-404.
Hviid 2018	Hviid A, Svanström H, Scheller NM, Grönlund O, Pasternak B, Arnheim-Dahlström L. Human papillomavirus vaccination of adult women and risk of autoimmune and neurological diseases. J Intern Med. 2018 Feb;283(2):154-165.
Chang 2019	Chang KH, Lyu RK, Lin WT, Huang YT, Lin HS, Chang SH. Gulllain-Barre Syndrome After Trivalent Influenza Vaccination in Adults. Front Neurol. 2019 Jul 24;10:768.
Chao 2012	Chao C, Klein NP, Velicer CM, Sy LS, Slezak JM, Takhar H, Ackerson B, Cheetham TC, Hansen J, Deosaransingh K, Emery M, Liaw KL, Jacobsen SJ. Surveillance of autoimmune conditions following routine use of quadrivalent human papillomavirus vaccine. J Intern Med. 2012 Feb;271(2):193-203.
Chen 2018	Chen Y, Ma F, Xu Y, Chu X, Zhang J. Vaccines and the risk of acute disseminated encephalomyelitis. Vaccine. 2018 Jun 18;36(26):3733-3739.
Juurlink 2006	Juurlink DN, Stukel TA, Kwong J, Kopp A, McGeer A, Upshur RE, Manuel DG, Moineddin R, Wilson K. Guillain-Barré syndrome after influenza vaccination in adults: a population-based study. Arch Intern Med. 2006 Nov 13;166(20):2217-21.
Kaplan 1982	Kaplan JE, Katona P, Hurwitz ES, Schonberger LB. Guillain-Barré syndrome in the United States, 1979-1980 and 1980-1981. Lack of an association with influenza vaccination. JAMA. 1982 Aug 13;248(6):698-700.

Karavanaki 2008	Karavanaki K, Tsoka E, Karayianni C, Petrou V, Pippidou E, Brisimitzi M, Mavrikiou M, Kakleas K, Konstantopoulos I, Manoussakis M, Dacou-Voutetakis C. Prevalence of allergic symptoms among children with diabetes mellitus type 1 of different socioeconomic status. Pediatr Diabetes. 2008 Aug;9(4 Pt 2):407-16.
Karvonen 1999	Karvonen M, Cepaitis Z, Tuomilehto J. Association between type 1 diabetes and Haemophilus influenzae type b vaccination: birth cohort study. BMJ. 1999 May 1;318(7192):1169-72.
Kawai 2014	Kawai AT, Li L, Kulldorff M, Vellozzi C, Weintraub E, Baxter R, Belongia EA, Daley MF, Jacobsen SJ, Naleway A, Nordin JD, Lee GM. Absence of associations between influenza vaccines and increased risks of seizures, Guillain-Barré syndrome, encephalitis, or anaphylaxis in the 2012-2013 season. Pharmacoepidemiol Drug Saf. 2014 May;23(5):548-53.
Klein 2019	Klein NP, Goddard K, Lewis E, Ross P, Gee J, DeStefano F, Baxter R. Long term risk of developing type 1 diabetes after HPV vaccination in males and females. Vaccine. 2019 Mar 28;37(14):1938-1944.
Koepsell 2010	Koepsell TD, Longstreth WT, Ton TG. Medical exposures in youth and the frequency of narcolepsy with cataplexy: a population-based case-control study in genetically predisposed people. J Sleep Res. 2010 Mar;19(1 Pt 1):80-6.
Kurtzke 1997	Kurtzke JF, Hyllested K, Arbuckle JD, Baerentsen DJ, Jersild C, Madden DL, Olsen A, Sever JL. Multiple sclerosis in the Faroe Islands. IV. The lack of a relationship between canine distemper and the epidemics of MS. Acta Neurol Scand. 1988 Dec;78(6):484-500.
Kwong 2013	Kwong JC, Vasa PP, Campitelli MA, Hawken S, Wilson K, Rosella LC, Stukel TA, Crowcroft NS, McGeer AJ, Zinman L, Deeks SL. Risk of Guillain-Barré syndrome after seasonal influenza vaccination and influenza health-care encounters: a self-controlled study. Lancet Infect Dis. 2013 Sep;13(9):769-76.
Lafaurie 2018	Lafaurie M, Baricault B, Lapeyre-Mestre M, Sailler L, Sommet A, Moulis G. Risk of Vaccine-Induced Immune Thrombocytopenia in Children. Nationwide Case Cross-over and Self-Controlled Case Series Studies in France. Blood 2018 Dec; 132 (supp 1): 738
Lai 2015	Lai YC, Yew YW. Severe Autoimmune Adverse Events Post Herpes Zoster Vaccine: A Case-Control Study of Adverse Events in a National Database. J Drugs Dermatol. 2015 Jul;14(7):681-4.
Lane 2003	Lane SE, Watts RA, Bentham G, Innes NJ, Scott DG. Are environmental factors important in primary systemic vasculitis? A case-control study. Arthritis Rheum. 2003 Mar;48(3):814-23.
Langer-Gould 2014	Langer-Gould A, Qian L, Tartof SY, Brara SM, Jacobsen SJ, Beaber BE, Sy LS, Chao C, Hechter R, Tseng HF. Vaccines and the risk of multiple sclerosis and other central nervous system demyelinating diseases. JAMA Neurol. 2014 Dec;71(12):1506-13.
Lasky 1998	Lasky T, Terracciano GJ, Magder L, Koski CL, Ballesteros M, Nash D, Clark S, Haber P, Stolley PD, Schonberger LB, Chen RT. The Guillain-Barré syndrome and the 1992-1993 and 1993-1994 influenza vaccines. N Engl J Med. 1998 Dec 17;339(25):1797-802.
Layton 2018	Layton JB, Butler AM, Panozzo CA, Brookhart MA. Rotavirus vaccination and short-term risk of adverse events in US infants. Paediatr Perinat Epidemiol. 2018 Sep;32(5):448-457.
Liang 2012	Liang JH. Review on domestic published papers regarding the acute disseminated encephalomyelitis and its relations with rabies vaccination. Zhonghua Liu Xing Bing Xue Za Zhi. 2012 Nov;33(11):1189-92.
Liu 2003	Liu GF, Wu ZL, Wu HS, Wang QY, Zhao-Ri GT, Wang CY, Liang ZX, Cui SL, Zheng JD. A case-control study on children with Guillain-Barre syndrome in North China. Biomed Environ Sci. 2003 Jun;16(2):105-11.
Liu 2018	Liu EY, Smith LM, Ellis AK, Whitaker H, Law B, Kwong JC, Farrington P, Lévesque LE. Quadrivalent human papillomavirus vaccination in girls and the risk of autoimmune disorders: the Ontario Grade 8 HPV Vaccine Cohort Study. CMAJ. 2018 May 28;190(21):E648-E
Loughlin 2012	Loughlin J, Mast TC, Doherty MC, Wang FT, Wong J, Seeger JD. Postmarketing evaluation of the short-term safety of the pentavalent rotavirus vaccine. Pediatr Infect Dis J. 2012 Mar;31(3):292-6.
Mahmud 2018	Mahmud SM, Bozat-Emre S, Mostaēo-Guidolin LC, Marrie RA. Registry Cohort Study to Determine Risk for Multiple Sclerosis after Vaccination for Pandemic Influenza A(H1N1) with Arepanrix, Manitoba, Canada. Emerg Infect Dis. 2018 Jul;24(7):1267-1274.
Malli 2015	Malli C, Pandit L, D'Cunha A, Mustafa S. Environmental factors related to multiple sclerosis in Indian population. PLoS One. 2015 Apr 22;10(4):e0124064.
McCarthy 2013	McCarthy NL, Gee J, Lin ND, Thyagarajan V, Pan Y, Su S, Turnbull B, Chan KA, Weintraub E. Evaluating the safety of influenza vaccine using a claims-based health system. Vaccine. 2013 Dec 5;31(50):5975-82.
McMahon 1992	McMahon BJ, Helminiak C, Wainwright RB, Bulkow L, Trimble BA, Wainwright K. Frequency of adverse reactions to hepatitis B vaccine in 43,618 persons. Am J Med. 1992 Mar;92(3):254-6.
Mikaeloff 2007 A	Mikaeloff Y, Caridade G, Rossier M, Suissa S, Tardieu M. Hepatitis B vaccination and the risk of childhood-onset multiple sclerosis. Arch Pediatr Adolesc Med. 2007 Dec;161(12):1176-82.
Mikaeloff 2009	Mikaeloff Y, Caridade G, Suissa S, Tardieu M. Hepatitis B vaccine and the risk of CNS inflammatory demyelination in childhood. Neurology. 2009 Mar 10;72(10):873-80.
Miranda 2017	Miranda S, Chaignot C, Collin C, Dray-Spira R, Weill A, Zureik M. Human papillomavirus vaccination and risk of autoimmune diseases: A large cohort study of over 2million young girls in France. Vaccine. 2017 Aug 24;35(36):4761-4768.
Montgomery 2002	Montgomery SM, Ehlin AG, Ekbom A, Wakefield AJ. Pertussis infection in childhood and subsequent type 1 diabetes mellitus. Diabet Med. 2002 Dec;19(12):986-93.
Morales-Sįnchez 2010	Morales-Sįnchez MA, Domķnguez-Gómez MA, Jurado-Santa Cruz F, Peralta-Pedrero ML. Immunization and bacterial pathogens in the oropharynx as risk factors for alopecia areata. Actas Dermosifiliogr. 2010 Jun;101(5):437-43.

Morris 2000	Morris DL, Montgomery SM, Thompson NP, Ebrahim S, Pounder RE, Wakefield AJ. Measles vaccination and inflammatory bowel disease: a national British Cohort Study. Am J Gastroenterol. 2000 Dec;95(12):3507-12.
Mouchet 2018	Mouchet J, Bégaud B. Central Demyelinating Diseases after Vaccination Against Hepatitis B Virus: A Disproportionality Analysis within the VAERS Database. Drug Saf. 2018 Aug;41(8):767-774.
Naleway 2009	Naleway AL, Belongia EA, Donahue JG, Kieke BA, Glanz JM; Vaccine Safety Datalink. Risk of immune hemolytic anemia in children following immunization. Vaccine. 2009 Dec 9;27(52):7394-7.
O'Leary 2012	O'Leary ST, Glanz JM, McClure DL, Akhtar A, Daley MF, Nakasato C, Baxter R, Davis RL, Izurieta HS, Lieu TA, Ball R. The risk of immune thrombocytopenic purpura after vaccination in children and adolescents. Pediatrics. 2012 Feb;129(2):248-55.
Oberle 2017	Oberle D, Pavel J, Mayer G, Geisler P, Keller-Stanislawski B. Retrospective multicenter matched case-control study on the risk factors for narcolepsy with special focus on vaccinations (including pandemic influenza vaccination) and infections in Germany. Sleep Med. 2017 Jun;34:71-83.
Ozakbas 2006	Ozakbas S, Idiman E, Yulug B, Pakoz B, Bahar H, Gulay Z. Development of multiple sclerosis after vaccination against hepatitis B: a study based on human leucocyte antigen haplotypes. Tissue Antigens. 2006 Sep;68(3):235-8.
Parent 1997	Parent ME, Siemiatycki J, Menzies R, Fritschi L, Colle E. Bacille Calmette-Guérin vaccination and incidence of IDDM in Montreal, Canada. Diabetes Care. 1997 May;20(5):767-72.
Pattison 2008	Pattison E, Harrison BJ, Griffiths CE, Silman AJ, Bruce IN. Environmental risk factors for the development of psoriatic arthritis: results from a case-control study. Ann Rheum Dis. 2008 May;67(5):672-6.
Payne 2006	Payne DC, Rose CE Jr, Kerrison J, Aranas A, Duderstadt S, McNeil MM. Anthrax vaccination and risk of optic neuritis in the United States military, 1998-2003. Arch Neurol. 2006;63(6):871-5.
Pekmezovic 2004	Pekmezovic T, Jarebinski M, Drulovic J. Childhood infections as risk factors for multiple sclerosis: Belgrade case-control study. Neuroepidemiology. 2004;23(6):285-8.
Perrett 2019	Perrett KP, Jachno K, Nolan TM, Harrison LC. Association of Rotavirus Vaccination With the Incidence of Type 1 Diabetes in Children. JAMA Pediatr. 2019;173(3):280-282
Piram 2016	Piram M, Madhi F, Ulinski T, Mahr A. Vaccination and Risk of Childhood IgA Vasculitis (Henoch–Schönlein): A Case-Crossover Analysis [abstract]. Arthritis Rheumatol. 2016; 68 (suppl 10). https://acrabstracts.org/abstract/vaccination-and-risk-of-childhood-iga-vasculitis-henoch-schonlein-a-case-crossover-analysis/. Accessed January 23, 2020.
Ramagopalan 2009	Ramagopalan SV, Valdar W, Dyment DA, DeLuca GC, Yee IM, Giovannoni G, Ebers GC, Sadovnick AD. Association of infectious mononucleosis with multiple sclerosis. A population-based study. Neuroepidemiology. 2009;32(4):257-62.
Rami 1999	Rami B, Schneider U, Imhof A, Waldhör T, Schober E. Risk factors for type I diabetes mellitus in children in Austria. Eur J Pediatr. 1999 May;158(5):362-6.
Ray 2011	Ray P, Black S, Shinefield H, Dillon A, Carpenter D, Lewis E, Ross P, Chen RT, Klein NP, Baxter R. Risk of rheumatoid arthritis following vaccination with tetanus, influenza and hepatitis B vaccines among persons 15-59 years of age. Vaccine. 2011 Sep 2;29(38):6592-7.
Rogers 2019	Rogers MAM, Basu T, Kim C. Lower Incidence Rate of Type 1 Diabetes after Receipt of the Rotavirus Vaccine in the United States, 2001-2017. Sci Rep. 2019 Jun 13;9(1):7727.
Rousseau 2016	Rousseau MC, El-Zein M, Conus F, Legault L, Parent ME. Bacillus Calmette-Guérin (BCG) Vaccination in Infancy and Risk of Childhood Diabetes. Paediatr Perinat Epidemiol. 2016 Mar;30(2):141-8.
Rowhani-Rahbar 2012	Rowhani-Rahbar A, Klein NP, Lewis N, Fireman B, Ray P, Rasgon B, Black S, Klein JO, Baxter R. Immunization and Bell's palsy in children: a case-centered analysis. Am J Epidemiol. 2012 May 1;175(9):878-85.
Sanghani 2018	Sanghani N, Hanumanthu R, Shah S, Souayah N. Myasthenia Gravis after Vaccination in Adults the United States: A Report from the CDC/FDA Vaccine Adverse Event Reporting System (1990–2017) (P6.437). Neurology 2018 Apr, 90 (15 Supplement) P6.437
Scanzi 2017	Scanzi F, Andreoli L, Martinelli M, Taraborelli M, Cavazzana I, Carabellese N, Ottaviani R, Allegri F, Franceschini F, Agmon-Levin N, Shoenfeld Y, Tincani A. Are the autoimmune/inflammatory syndrome induced by adjuvants (ASIA) and the undifferentiated connective tissue disease (UCTD) related to each other? A case-control study of environmental exposures. Immunologic Research 2017, 65 (1): 150-156
Shaw 2012	Shaw SY, Blanchard JF, Bernstein CN. Early childhood immunizations are not associated with pediatric IBD: a population-based analysis. Gastroenterology 2012 May;142(Suppl 1):S-88.
Shaw 2015	Shaw SY, Blanchard JF, Bernstein CN. Early childhood measles vaccinations are not associated with paediatric IBD: a population-based analysis. J Crohns Colitis. 2015 Apr;9(4):334-8.
Scheller 2015	Scheller NM, Svanström H, Pasternak B, Arnheim-Dahlström L, Sundström K, Fink K, Hviid A. Quadrivalent HPV vaccination and risk of multiple sclerosis and other demyelinating diseases of the central nervous system. JAMA. 2015 Jan 6;313(1):54-61.
Skrodenienė 2010	Skrodenienė E, Marčiulionytė D, Padaiga Ž, Jašinskienė E, Sadauskaitė-Kuehne V, Sanjeevi CB, Vitkauskienė A, Ludvigsson J. Associations between HLA class II haplotypes, environmental fac- tors and type 1 diabetes mellitus in Lithuanian children with type 1 diabetes and controls. Polish Ann Med 2010; 17 (1):7–15
Skufca 2018	Skufca J, Ollgren J, Artama M, Ruokokoski E, Nohynek H, Palmu AA. The association of adverse events with bivalent human papilloma virus vaccination: A nationwide register-based cohort study in Finland. Vaccine. 2018 Sep 18;36(39):5926-5933.

Sridhar, 2017	Sridhar G, Tian F, Forshee R, Kulldorff M, Selvam N, Sutherland A, Bryan W, Barone S, Xu L, Izurieta HS. Evaluation of optic neuritis following human papillomavirus vaccination. Hum Vaccin Immunother. 2017 Jul 3;13(7):1705-1713.
Stowe 2009	Stowe J, Andrews N, Wise L, Miller E. Investigation of the temporal association of Guillain-Barré syndrome with influenza vaccine and influenzalike illness using the United Kingdom General Practice Research Database. Am J Epidemiol 2009; 169: 382–88.
Stricker 1994	Stricker BH, van der Klauw MM, Ottervanger JP, van der Meché FG. A case-control study of drugs and other determinants as potential causes of Guillain-Barré syndrome. J Clin Epidemiol. 1994 Oct;47(10):1203-10.
Sturkenboom 1999	Sturkenboom MCJM, Abenhaim L, Wolfson C, Roulet E, Heinzlf O, Gout O. Vacination, demyelination, and multiple sclerosis. Pharmacoepidemiology and Drug Safety 1999; 8: S170–171.
Šipetić 2003	Sipetić S, Vlajinac H, Kocev N, Radmanović S. The belgrade childhood diabetes study: association of infections and vaccinations on diabetes in childhood. Ann Epidemiol. 2003 Oct;13(9):645-51.
Šipetić 2005	Sipetić SB, Vlajinac HD, Kocev NI, Marinković JM, Radmanović SZ, Bjekić MD. The Belgrade childhood diabetes study: a multivariate analysis of risk determinants for diabetes. Eur J Public Health. 2005 Apr;15(2):117-22.
Tam 2007	Tam CC, O'Brien SJ, Petersen I, Islam A, Hayward A, Rodrigues LC. Guillain-Barré syndrome and preceding infection with campylobacter, influenza and Epstein-Barr virus in the general practice research database. PLoS One. 2007 Apr 4;2(4):e344.
Telahun 1994	Telahun M, Abdulkadir J, Kebede E. The relation of early nutrition, infections and socio-economic factors to the development of childhood diabetes. Ethiop Med J. 1994 Oct;32(4):239-44.
Thompson 1995	Thompson NP, Montgomery SM, Pounder RE, Wakefield AJ. Is measles vaccination a risk factor for inflammatory bowel disease? Lancet. 1995 Apr 29;345(8957):1071-4.
Touze 2002	Touzé E, Fourrier A, Rue-Fenouche C, Rondé-Oustau V, Jeantaud I, Bégaud B, Alpérovitch A. Hepatitis B vaccination and first central nervous system demyelinating event: a case-control study. Neuroepidemiology. 2002 Jul-Aug;21(4):180-6.
Treadwell 2002	Treadwell TA, Maddox RA, Holman RC, Belay ED, Shahriari A, Anderson MS, Burns J, Glodé MP, Hoffman RE, Schonberger LB. Investigation of Kawasaki syndrome risk factors in Colorado. Pediatr Infect Dis J. 2002 Oct;21(10):976-8.
Vaarala 2017	Vaarala O, Jokinen J, Lahdenkari M, Leino T. Rotavirus Vaccination and the Risk of Celiac Disease or Type 1 Diabetes in Finnish Children at Early Life. Pediatr Infect Dis J. 2017 Jul;36(7):674-675.
Vahedi 2008	Vahedi H., Hojjatolah R, Fatemeh E, Malekzadeh R. Can Breast Feeding and Measles Vaccination in Childhood be Considered as Risk Factors for Later Inflammatory Bowel Diseases? Govaresh J. 2008 13(2): 81-88.
Vcev 2015	Vcev A, Pezerovic D, Jovanovic Z, Nakic D, Vcev I, Majnarić L. A retrospective, case-control study on traditional environmental risk factors in inflammatory bowel disease in Vukovar-Srijem County, north-eastern Croatia, 2010. Wien Klin Wochenschr. 2015 May;127(9-10):345-54.
Velentgas 2012	Velentgas P, Amato AA, Bohn RL, Chan KA, Cochrane T, Funch DP, Dashevsky I, Duddy AL, Gladowski P, Greenberg SA, Kramer JM, McMahill-Walraven C, Nakasato C, Spettell CM, Syat BL, Wahl PM, Walker AM, Zhang F, Brown JS, Platt R. Risk of Guillain-Barré syndrome after meningococcal conjugate vaccination. Pharmacoepidemiol Drug Saf. 2012 Dec;21(12):1350-8.
Verge 1994	Verge CF, Howard NJ, Irwig L, Simpson JM, Mackerras D, Silink M. Environmental factors in childhood IDDM. A population-based, case-control study. Diabetes Care. 1994 Dec;17(12):1381-9.
Verstraeten 2008	Verstraeten T, Descamps D, David MP, Zahaf T, Hardt K, Izurieta P, Dubin G, Breuer T. Analysis of adverse events of potential autoimmune aetiology in a large integrated safety database of AS04 adjuvanted vaccines. Vaccine. 2008 Dec 2;26(51):6630-8.
Villumsen 2013	Villumsen M, Jess T, Sørup S, Ravn H, Sturegård E, Benn CS, Aaby P, Roth A. Risk of inflammatory bowel disease following Bacille Calmette-Guérin and smallpox vaccination: a population-based Danish case-cohort study. Inflamm Bowel Dis. 2013 Jul;19(8):1717-24.
Willame 2016	Willame C, Rosillon D, Zima J, Angelo MG, Stuurman AL, Vroling H, et al. Risk of new onset autoimmune disease in 9- to 25-year-old women exposed to human papillomavirus-16/18 AS04-adjuvanted vaccine in the United Kingdom. Human Vacc Immunother 2016;12:286
Wise 2012	Wise ME, Viray M, Sejvar JJ, Lewis P, Baughman AL, Connor W, Danila R, Giambrone GP, Hale C, Hogan BC, Meek JI, Murphree R, Oh JY, Reingold A, Tellman N, Conner SM, Singleton JA, Lu PJ, DeStefano F, Fridkin SK, Vellozzi C, Morgan OW. Guillain-Barre syndrome during the 2009-2010 H1N1 influenza vaccination campaign: population-based surveillance among 45 million Americans. Am J Epidemiol. 2012 Jun 1;175(11):1110-9.
Yu 2007	Yu O, Bohlke K, Hanson CA, Delaney K, Rees TG, Zavitkovsky A, Ray P, Mullooly J, Black SB, Benson P, Thompson WW, Davis RL, Jackson LA. Hepatitis B vaccine and risk of autoimmune thyroid disease: a Vaccine Safety Datalink study. Pharmacoepidemiol Drug Saf. 2007 Jul;16(7):736-45.
Zilber 1996	Zilber N, Kahana E. Risk factors for multiple sclerosis: a case-control study in Israel. Acta Neurol Scand. 1996 Dec;94(6):395-403.
Zipp 1999	Zipp F, Weil JG, Einhäupl KM. No increase in demyelinating diseases after hepatitis B vaccination. Nat Med. 1999 Sep;5(9):964-5.
Zorzon 2003	Zorzon M, Zivadinov R, Nasuelli D, Dolfini P, Bosco A, Bratina A, Tommasi MA, Locatelli L, Cazzato G. Risk factors of multiple sclerosis: a case-control study. Neurol Sci. 2003 Nov;24(4):242-7.

Zou 2014	Zou YF, Feng CC, Zhu JM, Tao JH, Chen GM, Ye QL, Cen H, Leng RX, Pan FM, Pan HF, Li R, Fan YG, Wang B, Li XP, Zhang FY, Ye DQ. Prevalence of systemic lupus erythematosus and risk factors in rural areas of Anhui Province. Rheumatol Int. 2014 Mar;34(3):347-56.

Table S4. Descriptive information about and quality assessment of included studies.

Study: Author, Year	Country	Metho-dology	Women %	Age - range or median (years)	Total of patients	Vaccination	Autoimmune disorders	Follow-up/Time gap	Confounding/ Adjustment	NOS scale
Abbasi 2017	Iran	C-C	85	35.8	1081	Adult and childhood vaccination	MS	≥3 years	age, sex, education, ethnicity, income, and marital status.	4
Ahlgren 2009	Sweden	C-C	60.2	10-45	1094	MCV+RCV+MuCV	MS	≥3 years	unadjusted	7
Alter 1968	USA	C-C	NR	NR	108	TIV, DCV, TCV, SP, OPV/IPV, TF, wPCV, BCG	MS	≥3 years	unadjusted	4
Altobelli 2003	Italy	C-C	48.5	0-14	408	Childhood vaccination	T1D	≥3 years	unadjusted	6
Andersen 1981	Denmark	C-C	NR	30-50	324	BCG	MS	≥3 years	unadjusted	8
Andrews 2017	UK	SCCS/RI	100	11-19	101	HPV	GBS	90 days	possible time varying confounders of age in years, period	7
Angelo 2014	World	CT	100	9-72	41862	HPV	N, VIT, SLE, UC, CD, V, T1D, CCD, RA+JA, AA, HT, PSI, GD	1 year	unadjusted	5
Arnheim-Dahlström 2013	Denmark, Sweden	C	100	10-17	997585	HPV	SLE, BP, V, ON, PSI, BD, RA, CD, VIT, EN, HSP, T1D, PC, GD, CCD, JA, M, HT, SC, N, HT, RS, ITP, UC, AS	180 days	country, age in two year intervals, calendar year, and parental country of birth, parental education, and paternal socioeconomic status	8
Ascherio 2001	USA	C-C	100	30-55	724	HBV	MS	≥3 years	pack-years of smoking, latitude of residence at birth, history of infectious mononucleosis, history of measles or mumps after the age of 15, and ancestry	7
Baker 2019	USA	SCCS/RI	NR	0-2	87	PnCV	KS	28 days	age	9
Bansil 1997	India	C-C	61.9	34	147	DTP, SP, OPV, MMR, RAB, BCG	MS	≥3 years	unadjusted	4
Bardenheier 2016	USA	C-C	45.5	18-45+	345	TCV, HBV, MMR, HBV, TIV, TCV, TIV, MMR	SLE, RA	≥3 years	unadjusted	7
Baron 2005	France	C-C	49.3	0-17	564	MMR, SP, OPV/IPV, BCG	CD, UC	≥3 years	family history of IBD, breast feeding, history of eczema, and drinking tap water	8

Baxter 2013	USA	Cc	41.4	5-87	415	Any vaccination	GBS	42 days	age, sex, using cases from 1994 to 2005	7
Baxter 2016	USA	Cc	NR	NR	179	Any vaccination	ON	42 days	sex, age, vaccine	7
Belongia 2010	USA	C	NR	0-1	224228	RGEV	KS	30 days	age	7
Bengtsson 2010	Sweden	C-C	NR	18-70	3839	Any vaccination	RA	≥3 years	age, residential area and gender	7
Bernstein 2007	Canada	C-C	65.3	18-50	682	MMR	CD, UC	≥3 years	unadjusted	6
Berr 1989	France	C-C	73	30.8	126	OPV/IPV, BCG	MS	≥3 years	unadjusted	6
Bertuola 2010	Italy	C-C	46.2	0-18	2311	Any vaccination	ITP	42 days	age and use of drugs	6
Beyerlein 2017	Germany	SCCS/RI	45	0-10	1918	MenC, PnCV, VZV, MMR, DTaP-IPV-Hib-HBV, TBE, TIV	T1D	≥3 years	HLA genotype, sex, delivery mode, season of birth, preterm delivery and maternal T1D status	8
Black 2002	USA	C	NR	0-13	43978	Hib	T1D	≥3 years	unadjusted	5
Black 2003	USA	C-C	44.4	1-2	139	MMR	ITP	180 days	unadjusted	6
Block 2010	World	CT	100	9-26	21464	HPV	ON, JA, CCD, GD, MS, CD, HT, RA, VIT, RS, SLE, UC, T1D, PSI, TH, IBD, AA, EN, HT	≥3 years	unadjusted	5
Blom 1991	Sweden	C-C	46.7	0-14	867	RCV, TCV, OPV, BCG, SP, MCV, DTP, MuCV, DT	T1D	≥3 years	unadjusted	9
Cardwell 2008	UK	C-C	54.6	0-14	4946	DT-IPV, Hib, MMR, MenC, BCG, aPCV/wPCV	T1D	≥3 years	infections, vaccinations, antibiotics in the first year of life	8
Casetta 1994	Italy	C-C	64.6	46.5	254	YF, RAB, DCV, BCG, OPV/IPV, TCV, RCV	MS	≥3 years	unadjusted	6
Compston 1986	UK	C-C	60.7	32.2	341	TCV	MS, ON	≥3 years	unadjusted	2
Cooper 2002	USA	C-C	90.5	15-81	620	HBV	SLE	≥3 years	age, sex, state, race, and education	6
Da Dalt 2016	Italy	C-C	46	0-18	915	Any vaccination	HSP	84 days	age	4
Dauvilliers 2013	France	C-C	36.1	15.1	194	HBV, Hib, IPV, TCV, DCV, TIV, aPCV/wPCV, HPV	N	≥3 years	unadjusted	7
Davis 2001	USA	C-C	51.9	0-25	574	MCV	IBD	≥3 years	age, sex, race	7
Deceuninck 2018	Canada	C	100	9-17	947321	HPV	GBS	60 days	sex, age, year of GBS diagnosis and H1N1 pandemic period	4
DeStefano 2001	USA	C-C	43.5	0-11	1020	MMR, wPCV, VZV, aPCV, HBV, Hib	T1D	≥3 years	health maintenance organizations, length of enrollment, gender, date of birth, race/ethnicity and family history of possible type 1 diabetes.	7

DeStefano 2003	USA	C-C	76.6	18-49	1390	RCV, TIV, HBV, HAV, MCV, PSV, TCV	MS+ON	≥3 years	health maintenance organization, sex, date of birth, race, marital status, ever smoked, family history of a demyelinating disease, family history of autoimmune disease, place of birth, and Scandinavian ancestry	8
Duderstadt 2012	USA	C	8.3	17-35	2.4e+06	TF, MMR, HBV, SP, YF	T1D	≥3 years	age, race, sex, service branch, military grade, calendar year, and receipt of one or more of the study vaccines	7
Eftekharian 2014	Iran	C-C	73.4	33	454	HBV	MS	≥3 years	unadjusted	3
Feeney 1997	UK	C-C	NR	19	420	DT, aPCV/wPCV, MMR	IBD	≥3 years	unadjusted	7
France 2008	USA	SCCS/RI	47.6	1-2	63	MMR	ITP	42 days	age	9
Frisch 2018	Denmark	C	0	10-17	568410	HPV	PSI, T1D, JA, RA, UC, V, CCD, CD, VIT, AS, N, HT, BP, HSP	≥3 years	age, calendar period	8
Galeotti 2013	Italy	C-C	42.2	61	448	TIV	GBS	≥3 years	sex, age, region, admission date	7
Garbe 2012	Germany	C-C	51.9	18-92	2233	TIV	ITP	28 days	age, sex	6
Geier 2005	USA	C-C	76.8	>7	2178	HBV	SLE, AA, ON, ITP, RA, MS, V	≥3 years	unadjusted	5
Geier 2017	USA	C-C	100	NR	49036	HPV	ITP, AA, V, SLE, GBS, RA	≥3 years	unadjusted	4
Gilat 1987	World	C-C	49.7	20	1300	SP	CD	≥3 years	unadjusted	4
Glatthaar 1988	Australia	C-C	41.4	0-14	947	DT, OPV2, RCV, OPV1, DTP, MCV	T1D	≥3 years	unadjusted	9
Graves 1999	USA	C-C	47.9	0-12	317	OPV/IPV, HBV, DTP, Hib	T1D	≥3 years	unadjusted	7
Greene 2012	USA	SCCS/RI	62.5	2-83	14	TIV	GBS	42 days	age, sex, region, month of GBS onset	9
Grimaldi-Bensouda 2011	France	C-C	39	4-76	1225	TIV	GBS	180 days	unadjusted	9
Grimaldi-Bensouda 2012	France	C-C	60.1	18-79	1076	TIV, DTP+	ITP	1 year	family history of autoimmune disorders, systemic NSAID(s) in the 12 mo before the index date, vaccination with any vaccine other than influenza in the 24 mo before the index date	9

**Grimaldi-Bensouda 2014**	France	C-C	100	14-26	1365	HPV	T1D, GBS, CTD, ADS, ITP	2 years	risk score and a personal or family history of autoimmune disorders	8
**Grimaldi-Bensouda 2017**	France	C-C	100	11-25	2463	HPV	T1D, ITP, GBS, TH, ADS, CTD	2 years	age, familial/personal history of autoimmune disease, parent's place of birth, and use of any oral contraceptives or vaccines	9
**Grimaldi-Bensouda A 2014**	France	C-C	88	13-60	817	HBV, DTP+, TIV	SLE	2 years	smoking, alcohol consumption, pregnancy in the 24 months before the index date, family history of autoimmune disorders, number of medications taken in the 12 months before the index date, and medication potentially associated with the induction of SLE in the 24 months before the index date	7
**Gronlund 2016**	Sweden	C	100	10-30	70265	HPV	UN	180 days	age, country of birth, parental country of birth, parental income level, parental education level, extended risk period	8
**Hansen 2011**	Denmark	C-C	53.6	10-95	534	DCV, TCV, aPCV/wPCV, RCV, BCG, MCV, OPV/IPV	IBD	≥3 years	unadjusted	5
**Hapfelmeier 2019**	Germany	C-C	69.4	39.5	91447	Any vaccination	MS	≥3 years	age, sex	9
**Harrison 1997**	UK	C-C	65	16-88	343	Any vaccination	IP	42 days	social class, smoking	4
**Hernán 2004**	UK	C-C	69.4	36	1767	TIV, TCV, HBV	MS	3 years	smoking, sex, age, calendar time, clinical course of the disease, type of first symptoms	8
**Ho 2012**	Taiwan	C	52.4	75	93049	TIV	MS, BP, RA, AA, GBS, T1D	1 year	age	8
**Hughes 2006**	UK	SCCS/RI	46.9	0-100	228	Any vaccination	GBS	42 days	age, sex	8
**Hviid 2004**	Denmark	C	NR	0-12	723273	OPV, DTaP-IPV, DT-IPV,	T1D	≥3 years	sex, age, calendar period	9

						Hib, wPCV, MMR				
Hviid 2018	Denmark +Sweden	C	100	18-44	3.1e+06	HPV	CD, HT, PSI, PC, RA, GD, AIHA, CCD, BD, AD, VIT, UC, AS, HT, WG, EN, SC, BP, SLE, RS, GSS, T1D, HT, HSP, PA, MG, ITP, M, N, V	≥3 years	age, calendar year, country of residence	9
Chang 2019	Taiwan	C-C	37.9	65.2	1092	TIV	GBS	90 days	unadjusted	6
Chao 2012	USA	C	100	9-26	529912	HPV	U, GD, SLE, HT, JA, ITP, RA, T1D, MS, ON	180 days	unadjusted	7
Chen 2018	China	C-C	45	0-61	1368	HAV, RAB, OPV, MMR, VZV, JE, HBV, TIV, MenC, DTaP	ADEM	180 days	nationality, occupation, marital status, allergy, familial diseases, comorbid chronic diseases, and history of infectious diseases within 6 months before the index date	8
Juurlink 2006	Canada	SCCS/RI	NR	20	269	TIV	GBS	49 days	sex, age, region	8
Kaplan 1982	USA	C	43	>18	1.7e+08	TIV	GBS	56 days	unadjusted	6
Karavanaki 2008	Greece	C-C	44.2	2-16	203	MMR	T1D	≥3 years	unadjusted	4
Karvonen 1999	Finland	C	NR	0-10	245288	Hib	T1D	≥3 years	unadjusted	5
Kawai 2014	USA	SCCS/RI	43.6	>0.5	116	TIV	GBS	42 days	age, site	7
Klein 2019	USA	C	54.8	11-35	911648	HPV	T1D	≥3 years	sex, race, medicaid status, years of prior KPNC membership	8
Koepsell 2010	USA	C-C	63	18-50	140	DCV, RCV, aPCV/wPCV, VZV, MCV, OPV/IPV, HBV, MuCV	N	≥3 years	race, annual income	7
Kurtzke 1997	Faroe Islands	C-C	51.3	0-30	150	YF, aPCV/wPCV, DCV, SP, MuCV, OPV/IPV, TF, TCV	MS	≥3 years	unadjusted	4
Kwong 2013	Canada	SCCS/RI	45.8	0-80	330	TIV	GBS	42 days	unadjusted	8
Lafaurie 2018	France	CCo	46.5	0-18	2459	MMR, MenC, DTaP-IPV-Hib-HBV, PnCV, DTaP-IPV, DT-IPV	ITP	42 days	exposure to other drugs known as inducers of ITP, seasonality	7
Lai 2015	USA	C-C	64.7	65	1243	HZV	AA, MS+ON, ITP, V, SLE, GBS	≥3 years	unadjusted	4
Lane 2003	UK	C-C	NR	17-89	295	Any vaccination	V	180 days	age, social class, urban/rural residence, job exposure, allergy, any vaccination within 6 months of the index date,	5

									steroid withdrawal within 6 months of the index date, smoking history	
Langer-Gould 2014	USA	C-C	69.3	2-86	4665	HPV, HBV	ADS	3 years	race/ethnicity, hospitalizations, outpatient visits, emergency department visits, comorbid chronic diseases, and infections within 6 months before the symptom onset/index date	7
Lasky 1998	USA	C	58	18-90	1.2e+08	TIV	GBS	42 days	age, sex, vaccine season	7
Layton 2018	USA	C	48.5	0-1	1.0e+06	RGEV	KS	30 days	sex, birth year, age	8
Liang 2012	China	C	NR	>0	4.1e+07	RAB	ADEM	≥3 years	age	3
Liu 2003	China	C-C	49	1-14	102	JE, OPV, TIV, HBV	GBS	≥3 years	unadjusted	5
Liu 2018	Canada	C	100	12-17	290939	HPV	UC, ITP, T1D, AIHA, CD, AIH, ADEM, JA, HT, GD, ON, BP	60 days	age, seasonality, receipt of non-HPV vaccines, recent infection	8
Loughlin 2012	USA	C	49	0-1	147767	RGEV	KS	30 days	unadjusted	8
Mahmud 2018	Canada	C	59.4	≥0.5	898074	TIV	MS	≥3 years	age	8
Malli 2015	India	C-C	66.2	37	417	Any vaccination	MS	≥3 years	unadjusted	3
McCarthy 2013	USA	SCCS/RI	NR	0-80	1021	TIV	ADS, GBS, BP	42 days	unadjusted	7
McMahon 1992	USA	C	NR	20-40	189410	HBV	GBS	1 year	unadjusted	5
Mikaeloff 2007 A	France	C-C	61.8	0-16	1265	HBV	MS	≥3 years	family history of MS or of other autoimmune diseases, profession of the head of the family	8
Mikaeloff 2009	France	C-C	54.3	0-16	3290	HBV	ADS	≥3 years	familial multiple sclerosis history, family history of another autoimmune disease, parental smoking at home before index date, socioprofessional status of the head of the family	8
Miranda 2017	France	C	100	13-16	2.3e+06	HPV	SLE, PC, ADS, RA+JA, T1D, GSS, ITP, IBD, GBS, TH, SC, V, CCD, M	≥3 years	year of inclusion, geographical zone, CMUc, history of use of health care	8

Montgomery 2002	UK	C	48.5	30	12519	wPCV, TCV, SP	T1D	≥3 years	and other vaccinations, use of health care and other vaccinations after inclusion age of pertussis infection, number of pertussis vaccinations by age, wild measles, mumps and chickenpox infections; tetanus and smallpox immunizations , sex, social class and age	8
Morales-Sánchez 2010	Mexico	C-C	NR	18-59	130	Any vaccination	AA	180 days	unadjusted	4
Morris 2000	UK	C	51.9	0-26	7319	MCV	T1D, IBD	≥3 years	sex, household crowding in childhood, fat her's social class at birth	7
Mouchet 2018	USA	C	NR	18-49	507052	HBV	MS	112 days	unadjusted	3
Naleway 2009	USA	SCCS/RI	56	0-17	55	Any vaccination	AIHA	42 days	unadjusted	8
O'Leary 2012	USA	SCCS/RI	49	0-17	197	PnCV, Hib, DTaP, HAV, TIV, MMR	ITP	42 days	unadjusted	9
Oberle 2017	Germany	C-C	62.4	5-55	367	TBE, HBV, OPV/IPV, aPCV/wPCV, HPV, DCV, TCV	N	≥3 years	unadjusted	6
Ozakbas 2006	Turkey	C-C	65.7	12-50	102	HBV	MS	56 days	unadjusted	4
Parent 1997	Canada	C-C	42.8	0-18	3676	BCG	T1D	≥3 years	unadjusted	8
Pattison 2008	UK	C-C	50.6	46-54	261	Any vaccination	PsA	≥3 years	age, gender	3
Payne 2006	USA	C-C	59.9	30.5	4524	SP, TIV, HBV	ON	<2years	sex, deployment, service component, previous multiple sclerosis diagnosis, race, ethnicity, age, military service branch, occupational group	8
Pekmezovic 2004	Serbia	C-C	70	35	220	SP, aPCV/wPCV, TCV, MuCV, DCV, OPV, BCG, MCV	MS	≥3 years	sex, age, area of residence	5
Perrett 2019	Australia	C	NR	0-4	2.8e+06	RGEV	T1D	≥3 years	unadjusted	5
Piram 2016	France	CCo	48	0-17	167	Any vaccination	HSP	90 days	sex, age	7
Ramagopalan 2009	Canada	C-C	58.2	50.4	22033	HBV, TIV, MuCV, MCV, RCV	MS	≥3 years	sex, age	6
Rami 1999	Austria	C-C	46.6	0-15	609	TBE, TCV, MuCV, MCV, TIV, OPV/IPV, DCV, aPCV/wPCV, RCV, BCG	T1D	≥3 years	unadjusted	8

Ray 2011	USA	C-C	61.5	13-64	1660	TIV, HBV, TCV	RA	2 years	sex, race, exact number of utilization visits	8
Rogers 2019	USA	C	48.7	0-11	786917	RGEV	T1D	≥3 years	sex, season of birth, region of the country	8
Rousseau 2016	Canada	C	48.9	17	78492	BCG	T1D	≥3 years	sex, BWGA, number of older siblings, maternal age at childbirth, family income, area of residence, parental place of birth, the presence of allergic diseases	9
Rowhani-Rahbar 2012	USA	Cc	60.5	0-18	233	Any vaccination	BP	28 days	unadjusted	8
Sanghani 2018	USA	CCo	47.9	18-84	71	Any vaccination	MG	42 days	unadjusted	6
Scanzi 2017	Italy	C-C	88	50	184	HBV, TCV	CTD	≥3 years	unadjusted	5
Shaw 2012	Canada	C-C	NR	0-18	951	DT, OPV/IPV	IBD	≥3 years	physician visits in the first 2 years of life	6
Shaw 2015	Canada	C-C	43	0-18	951	MCV	IBD	≥3 years	number of non gastrointestinal-related physician visits in the first 2 years of life, socio-economic factor index scores at case date	6
Scheller 2015	Denmark, Sweden	C	100	10-44	4.0e+06	HPV	MS	2 years	calendar year, age, country	8
Skrodenienė 2010	Lithuania	C-C	51.5	0-15	202	DTP	T1D	≥3 years	mother's residence during pregnancy in village or remote house, egg introduction before 5th month of age, varicella infection, rubella infection, infection during the last 6 months before diagnosis of T1D, stressful event previous 6 months before diagnosis	5
Skufca 2018	Finland	C	100	11-15	240605	HPV	CD, HSP, CCD, ITP, JA, T1D, TH, HT, EN, M, VIT, RS, SLE, PSI, GBS, SC, PC, GD, BP, UC, HT, PAN	3 years	geographical area, country of origin, number of hospital contacts from 9 through 10 years of age	9

**Sridhar, 2017**	USA	C	100	9-26	655836	HPV	ON	60 days	history of other autoimmune diseases, history of other vaccinations, enhanced Deyo-Charlson index, region	9
**Stowe 2009**	UK	SCCS/RI	43.1	>0	775	TIV	GBS	180 days	age, calendar month	9
**Stricker 1994**	Netherlands	C-C	48.4	47.6	434	Any vaccination	GBS	90 days	oral con traceptives, vaccines, fever, malaise, diarrhoea, gastroenteritis, coughing, pharyngitis, respiratory tract infection, influenza-like disease, urinary tract infections	6
**Sturkenboom 1999**	UK	C-C	NR	20-60	500	Any vaccination	AD	1 year	unadjusted	5
**Šipetić 2003**	Serbia	C-C	49.5	0-16	315	Any vaccination	T1D	≥3 years	father's age at the initiation, consumption of nitrosoamines-rich food and weight gain 15 kg by mother during pregnancy, birth order of child, stressful events and symptoms of psychological dysfunction during the 12 months preceding the onset of the disease, infect ions during the 6 months preceding the onset	6
**Šipetić 2005**	Serbia	C-C	49.5	0-16	315	Any vaccination	T1D	≥3 years	stressful events, psychological dysfunction, infections during 6 months preceding the onset, higher education level of father, use of ultrasound diagnostic technologies, mother's consumption nitrosoamines-rich foods during pregnancy, alc	6

									ohol consumption by father, type 1 diabetes among relatives, type 2 diabetes among relatives	
**Tam 2007**	UK	C-C	NR	NR	5998	TIV	GBS	60 days	sex, age, region	8
**Telahun 1994**	Ethiopia	C-C	59.2	1-15	140	MCV, BCG, DTP+	T1D	≥3 years	unadjusted	5
**The EURODIAB 2000**	Europe	C-C	NR	0-15	3202	BCG, RCV, aPCV/wPCV, TCV, OPV/IPV, MuCV, MCV, DCV, Hib	T1D	≥3 years	center, age-group, breast feeding, birth weight, maternal age, jaundice at birth, asthma before disease diagnosis, vitamin D supplementation	7
**Thompson 1995**	UK	C	NR	0-31	14952	MCV	CCD, UC, CD	≥3 years	unadjusted	7
**Touze 2002**	France	C-C	77.2	34	591	HBV	AD	1 year	unadjusted	8
**Treadwell 2002**	USA	C-C	NR	0-13	57	Any vaccination	KS	30 days	unadjusted	6
**Vaarala 2017**	Finland	C	NR	0-5	121650	RGEV	T1D, CCD	≥3 years	baseline rate in biannual cohorts	8
**Vahedi 2008**	Iran	C-C	53.8	NR	598	MCV	UC, CD	≥3 years	sex, education, matrimonial, rural/urban, smoking, breastfeeding	3
**Vcev 2015**	Croatia	C-C	48.7	>18	300	MCV	CD, UC	≥3 years	unadjusted	3
**Velentgas 2012**	USA	C	49.4	11-18	9.6e+06	MenC	GBS	≥3 years	unadjusted	9
**Verge 1994**	Australia	C-C	54.1	0-15	439	MuCV	T1D	≥3 years	maternal education level	6
**Verstraeten 2008**	World	CT	NR	10-87	68512	HPV	ON, PSI, TH, ITP, IBD, EN, RA, MS, CD, HT	≥3 years	unadjusted	5
**Villumsen 2013**	Denmark	C	49.7	0-44	47000	SP, BCG	CD	≥3 years	sex, year of birth, BCG and smallpox vaccination status, immigration status	9
**Willame 2016**	UK	C	100	9-25	129546	HPV	UC, ITP, RA, JA, T1D, CD, TH, PsA	1 year	unadjusted	6
**Wise 2012**	USA	C	48	>0	2.0e+07	TIV	GBS	42 days	age, sex	7
**Yu 2007**	USA	C-C	74.3	18-69	1875	HAV	HT, GD	≥3 years	age, sex, site, index year, personal and family history of autoimmune disease, smoking status, race, education	7
**Zilber 1996**	Israel	C-C	63	20-70	187	OPV, IPV, TCV, MCV, TF	MS	≥3 years	unadjusted	6
**Zipp 1999**	USA	C	50.8	>0	134698	HBV	AD	3 years	unadjusted	6
**Zorzon 2003**	Italy	C-C	64.3	17-65	271	BCG, OPV, MCV, MuCV, TIV, RCV, VZV	MS	≥3 years	socio-economic, environmental	5

Zou 2014	China	C-C	91.5	39.1	520	HBV	SLE	≥3 years	age, sex, familial variables	5

NR … not reported; Methodology: CT - clinical trials; C-C case-control study; C - cohort study; SCCS/RI - self- controlled case-series or self-controlled risk interval study; CCo - case cross-over study; CC - case-centered study; Vaccination: BCG - vaccine against tuberculosis; VZV - vaccine against chicken pox; DT - vaccine against diphtheria and tetanus; DT-IPV - vaccine against diphtheria, tetanus and polio; DTP - vaccine against diphtheria, tetanus, pertussis; DTaP - vaccine against diphtheria, tetanus, pertussis (acellular); DTaP-IPV - vaccine against diphtheria, tetanus, pertussis (acellular), polio; DTaP-IPV-Hib-HBV - vaccine against diphtheria, tetanus, pertussis (acellular), polio, Haemophilus influenzae type b and hepatitis B; DCV - diphtheria containing vaccine; HAV - vaccine against hepatitis A; HBV - vaccine against hepatitis B; HPV - vaccine against human papillomavirus; HZV - vaccine against herpes zoster; Hib - vaccine against Haemophilus influenzae type b; TIV - trivalent influenza vaccine; JE - vaccine against Japanese encephalitis; MMR - vaccine against measles, mumps and rubella; MCV - measles containing vaccine; MenC - vaccine against meningococcal serogroup C; MuCV - mumps containing vaccine; PSV - pneumococcal polysaccharide vaccine; aPCV - acellular pertussis vaccine; aPCV/wPCV - acellular or whole-cell pertussis vaccine; wPCV - whole-cell pertussis vaccine; PnCV - pneumococcal conjugated vaccine; IPV - inactivated polio vaccine; OPV - oral polio vaccine; OPV/IPV - oral or inactivated polio vaccine; OPV1 - oral polio vaccine serotype 1; OPV2 - oral polio vaccine serotype 2; RAB - vaccine against rabies; RGEV - vaccine against rotavirus gastroenteritis; RCV - rubella containing vaccine; SP - vaccine against smallpox; TBE - vaccine against tick-borne encephalitis; TCV - tetanus containing vaccine; TF - vaccine against typhoid fever; YF - vaccine against yellow fever; Autoimmune disorders: AA - Alopecia areata; AD - Addison's disease; ADEM - Acute disseminated encephalomyelitis; ADS - Acquired / Acute demyelinating syndrome; AIH - Autoimmune hepatitis; AIHA - Autoimmune haemolytic anemia; AS - Ankylosing spondylitis; BD - Behcet's disease; BP - Bell's palsy; CCD - Celiac disease; CD - Crohn's disease; EN - Erythema nodosum; GBS - Guillain-Barré syndrome; GD - Graves-Basedow's disease; GN – Glomerulonephritis; HSP - Henoch-Schönlein purpura; HT - Hashimoto's thyroiditis/Hyperthyroidism/Hypothyroidism; IBD - Inflammatory bowel disease; IP - Inflammatory polyarthritis; ITP - Immune thrombocytopenia / Idiopathic thrombocytopenic purpura; JA - Juvenile arthritis; KS - Kawasaki syndrome; M - Myositis, PsA - Psoriatic arthritis; MG - Myasthenia gravis; MS - Multiple sclerosis; N – Narcolepsy; ON - Optic neuritis; PA - Pernicious anemia; PAN - Polyarteritis nodosa; PC – Pancreatitis; PSI – Psoriasis; RA - Rheumatoid arthritis; RS - Raynaud's syndrome; SC - Scleroderma (local and / or systemic lupus); SjS - Sjögren's syndrome; SLE – Systemic lupus erythematosus; T1D - Type 1 diabetes; TH – Thyroiditis; U – Uveitis; UC - Ulcerative colitis; V – Vasculitis; VIT – Vitiligo; WG - Wegener's granulomatosis
